# Novel scaffold platforms for simultaneous induction osteogenesis and angiogenesis in bone tissue engineering: a cutting-edge approach

**DOI:** 10.1186/s12951-023-02115-7

**Published:** 2023-09-28

**Authors:** Arezoo Saberi, Maryam Kouhjani, Marzieh Mohammadi, Leticia Hosta-Rigau

**Affiliations:** 1 Department of Pharmaceutics, School of Pharmacy, Mashhad University of Medical Sciences, Mashhad, Iran; 2 DTU Health Tech, Centre for Nanomedicine and Theranostics, Technical University of Denmark, Produktionstorvet, Building 423, 2800 Kgs. Lyngby, Denmark

**Keywords:** Bone tissue engineering, Scaffold, Simultaneous osteogenesis and angiogenesis, Nanoparticles, Gene delivery

## Abstract

Despite the recent advances in the development of bone graft substitutes, treatment of critical size bone defects continues to be a significant challenge, especially in the elderly population. A current approach to overcome this challenge involves the creation of bone-mimicking scaffolds that can simultaneously promote osteogenesis and angiogenesis. In this context, incorporating multiple bioactive agents like growth factors, genes, and small molecules into these scaffolds has emerged as a promising strategy. To incorporate such agents, researchers have developed scaffolds incorporating nanoparticles, including nanoparticulate carriers, inorganic nanoparticles, and exosomes. Current paper provides a summary of the latest advancements in using various bioactive agents, drugs, and cells to synergistically promote osteogenesis and angiogenesis in bone-mimetic scaffolds. It also discusses scaffold design properties aimed at maximizing the synergistic effects of osteogenesis and angiogenesis, various innovative fabrication strategies, and ongoing clinical studies.

**Graphical Abstract:**

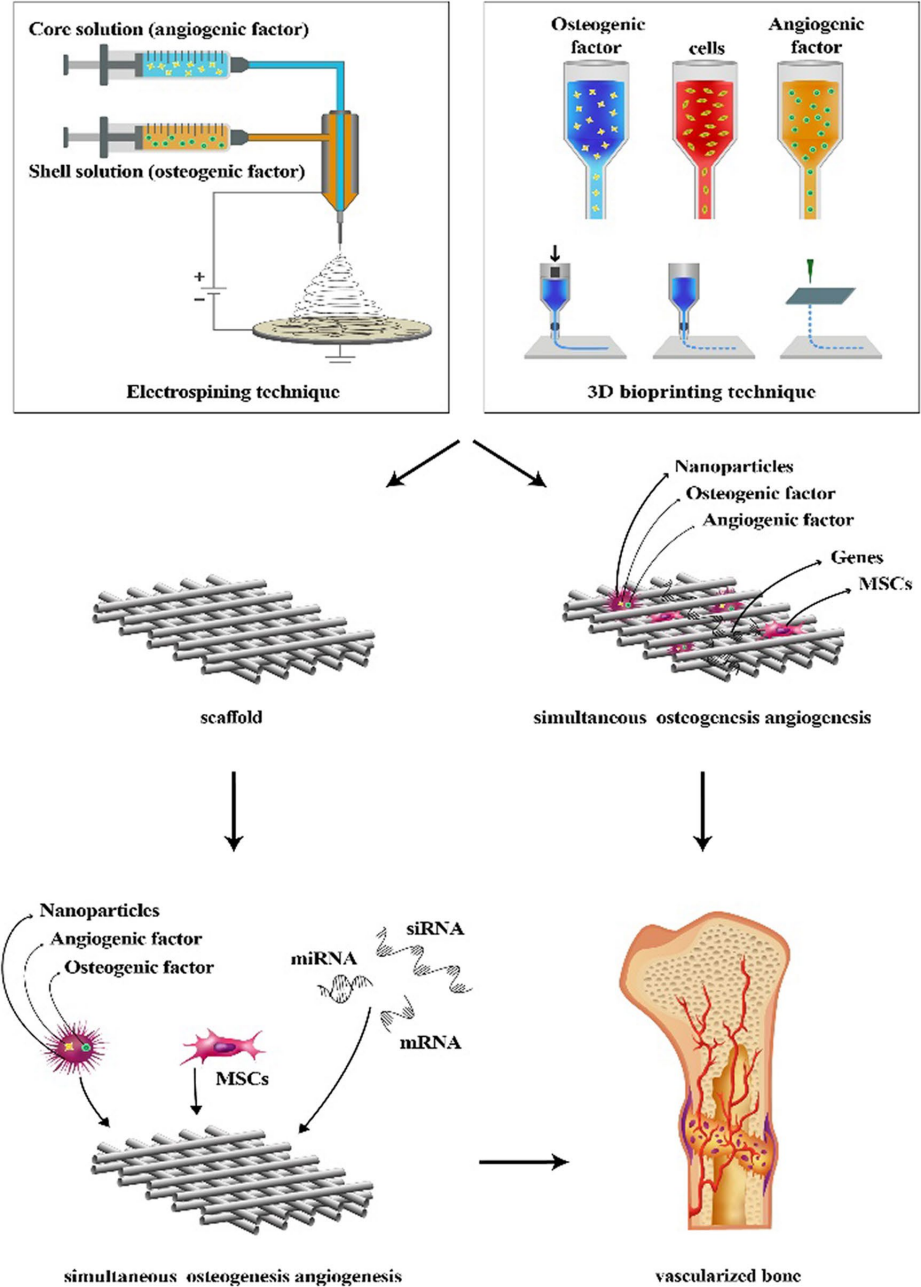

## Introduction

Critical bone defect reconstruction is a challenging procedure in orthopedics. Traumatic injuries, tumor resection, degenerative diseases such as osteoporosis and osteoarthritis are the major causes of bone defects leading to poor physical activity, severe pain, and deformities. The worldwide increase of elderly populations which leads to an increased chance of developing nonhealing fractures, has promoted an increased focus on developing functional bone graft substitutes [1–3].

Although bone possesses an intrinsic ability for regeneration, due to its complicated structure, the healing of critical bone defects remains challenging. Autologous bone grafts, which are nowadays the gold standard for bone repair, have important drawbacks such as serious donor site injury, associated morbidities, the need for a second surgery and up to 50% failure rates in certain sites [4, 5]. To overcome these limitations, research efforts have focused on the design and development of bone graft substitutes that mimic the structure and activity of natural bones.

Native bone is constituted by a combination of cells together with organic–inorganic matter arranged in a hierarchical design that includes nanoscale to micro level structures. Specifically, four different cell types (i.e., osteoclasts, osteocytes, osteoblasts (OBs), and bone lining cells which are all of them responsible for dynamic bone remodeling, are embedded within an extracellular matrix (ECM) composed of type 1 collagen, nano-hydroxy apatite (HA), proteoglycans and glycoproteins [6]. Moreover, at predetermined time points, growth factors (GFs), proteins and cytokines are released to regulate bone function [7]. GFs are a subgroup of soluble proteins that are extensively used to induce bone regeneration due to the key role that they play in the biological cascade of bone remodeling [8–10]. The most important GFs that regulate bone development include fibroblast growth factors (FGF), platelet-derived growth factors (PDGF), vascular endothelial growth factors (VEGF), insulin-like growth factors (IGF) and bone morphogenetic proteins (BMPs) [11].

In this context, the field of bone tissue engineering (BTE) aims at developing new strategies to render scaffold platforms that mimic not only the structure and composition of the ECM but also its functionality. Such a challenge is achieved by embedding a wide range of bioactive agents such as genetic materials, GFs, small molecules or drugs with controlled stimulation and release [12]. With such an approach, the differentiation of stem cells (SCs) into bone tissue is promoted and regulated [13]. Ideal platforms for BTE applications are scaffolds displaying a three-dimensional (3D) architecture that also offer sufficient surface-area-to-volume-ratio without compromising a balanced biodegradability and biomechanical strength. Importantly, the scaffolds should also support cellular adhesion, proliferation, and differentiation.

For a successful regulation of the differentiation process, sustained release is needed, and this is usually achieved by encapsulating the active compounds within predefined drug delivery vehicles. Furthermore, the presence of such carriers makes it possible not only to inhibit their burst release and the associated adverse effects, but also to enhance the loading capacity. When the administration of GFs is the aim, with the use of carriers, the half-life of the GFs and their biodistribution can be adjusted. In this regard, Tang et al. designed a biomimetic scaffold constituted by mesoporous bioactive glass (BG) and sulfated chitosan (CHI) that also incorporated BMP-2 and gelatin-methacryloyl (GelMA) hydrogel columns containing VEGF [14]. With such a scaffold, the authors were able to achieve a specific release pattern with an initial high liberation of VEGF followed by a decreasing concentration over time and a sustained release of BMP-2. The results showed that the as-prepared dual modular scaffold promoted osteogenic differentiation and capillary tube formation which, in turn, accelerated the regeneration of the bone tissue. Apart from the incorporation of GFs, drug delivery vehicles have also been employed as gene vectors that allow for gene condensation thus facilitating their transfection into the target cell and inducing the subsequent osteogenesis. In this regard, Raftery et al. designed a multi-cistronic plasmid encoding both BMP-2 and BMP-7 and used CHI nanoparticles (NPs) for the successful delivery to adjacent mesenchymal SCs (MSCs). The resulting plasmid-loaded NPs were subsequently incorporated into a collagen-HA scaffold to mimic the composition of mineral bone. With such an approach, the authors were able to demonstrate that the scaffold was able to promote the differentiation of osteoprogenitor cells as well as to enhance bone formation in a critical size defect rat model [15].

Despite the considerable advancements made in recent years, exemplified by the aforementioned examples, a significant obstacle persists in achieving successful osteogenesis. This challenge revolves around the need to ensure optimal delivery of oxygen and nutrients to the scaffold cells while effectively removing waste products [16]. For that, concurrent induction of osteogenesis but also angiogenesis is essential. Angiogenesis, that is, the formation of new blood vessels, is vital for cell survival and the integration of the implant with the bone tissue [17]. As such, inadequate bone vascularity results in nutritional limitations, cell death and reduced bone formation. In this context, several studies have demonstrated that, when angiogenesis is not induced, vascular infiltration into the engineered tissue is too slow to provide an acceptable blood supply. This highlights how bone formation and repair are closely related to the osteogenesis–angiogenesis interplay.

To this end, research efforts have moved the focus towards the development of functional scaffolds providing simultaneous osteogenesis and vascularization. To reach this goal, three main methodologies are currently being developed: (1) the assembly of NPs-eluting scaffolds to provide a sustained release pattern for GFs, small molecules or drugs; (2) the fabrication of gene-eluting scaffolds with improved transfection of the target cells; and (3) the development of multifunctional scaffolds with various cell types, genetic materials, drugs, and GFs.

The current review discusses the essential criteria to fabricate biomimetic scaffold platforms for BTE and introduces different types of scaffold platforms with concurrent induction of osteogenesis and angiogenesis. Current clinical studies trials are also summarized. In addition, various preparation technologies and methods to design and develop functional bone graft substitutes are mentioned.

## The process of bone repair

**Fig. 1:**
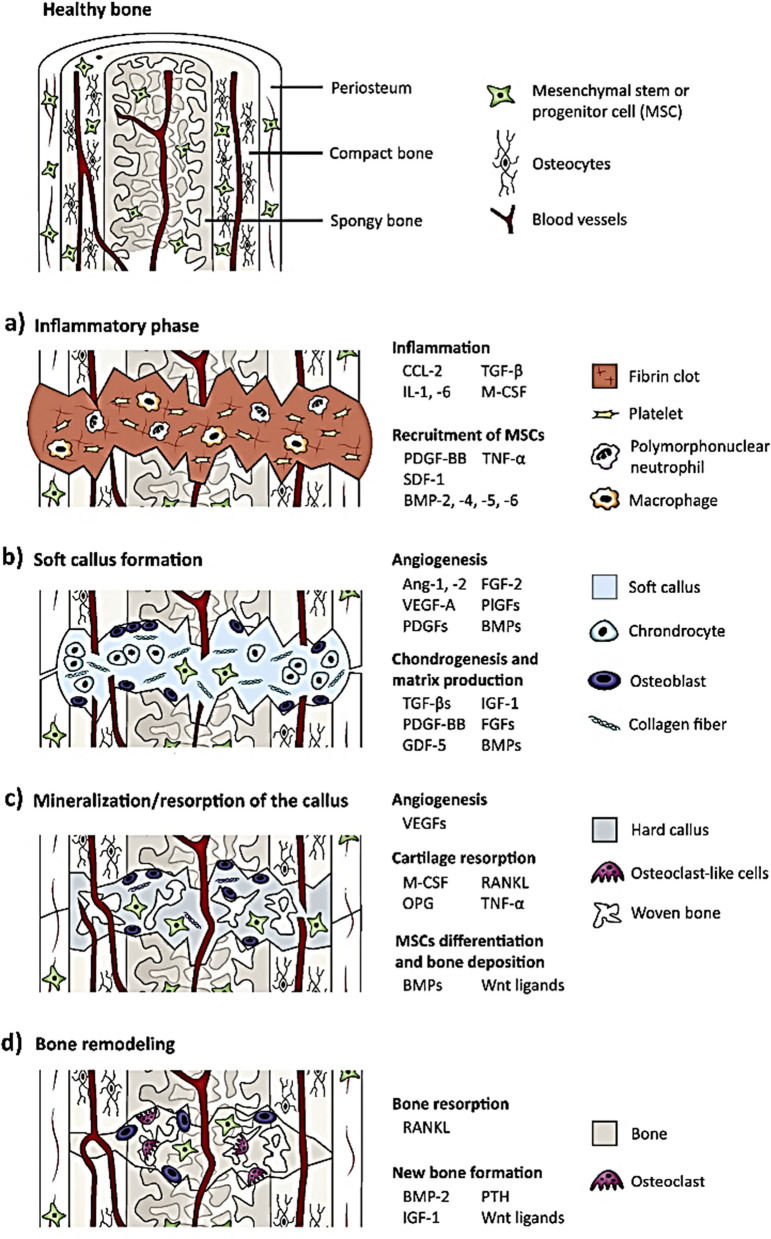
The bone repair process involves four phases: **a** inflammatory phase, **b** soft callus formation, **c** hard callus formation and **d** bone remodeling. Different growth factors and cytokines are secreted during each phase. SDF-1, stromal cell-derive factor 1; RANKL, receptor activator of nuclear factor κB ligand; VEGF, vascular endothelial growth factor; M-CSF, macrophage colony-stimulating factor; PlGF, placental growth factor; GDF-5, growth/differentiation factor 5; OPG, osteoprotegerin; BMP, bone morphogenetic protein; TNF-α, tumor necrosis factor α; PTH, parathyroid hormone; PDGF, platelet-derived growth factor; FGF, fibroblast growth factor. Adapted with permission from [21]

During bone repair, a variety of processes need to be coordinated and these can be grouped in four different stages: the inflammation response, the formation of the soft and the hard callus and the remodeling of the bone (Fig. 1) [18, 19]. Inflammation, which is the initial stage in bone healing and takes place during the first 24 h after an injury, involves the controlled release of several cytokines and GFs from the bone injury site [20]. During the second stage of bone repair, the formation of the soft callus takes place in a process that is dominated at the cellular level by chondrocytes and fibroblasts which provide mechanical support for the bone fracture. This is followed by the formation of the hard callus. At this stage the osteoblast activity is high, and the mineralized bone matrix is developed. Finally, the woven bone hard callus is remodeled into compact cortical bone [18].

During the inflammation stage, the levels of cytokines like interleukin (IL)-1, IL-6, tumor necrosis factor-α (TNF-α) and macrophage colony-stimulating factor as well as GFs like BMP or the transforming growth factor β (TGF-β), are considerably increased [22]. These factors signal the inflammatory cells (macrophages and neutrophils) and stimulate the differentiation of MSCs [23]. Amongst the different inflammatory cells, two types of macrophages (i.e., M1 and M2) play a prominent role in the biomaterial-related immune responses. Specifically, M1 macrophages boost inflammation through the release of inflammatory cytokines (i.e., IL-12 and IL-23) while, the M2 phenotype, is involved in improving tissue healing by releasing anti-inflammatory cytokines such as IL-10. Thus, being able to influence the macrophages phenotype (i.e., to induce the M2 phenotype) has emerged as a powerful tool to modulate the body response to the implant [24]. An example of this strategy is shown in a report by Weng et al. [25]. The authors were able to promote a transition from the M1 to the M2 macrophage phenotype involved in the inflammation process when using a 3D printed polycaprolactone (PCL)/nano-HA scaffold coated by a biomimetic glycopeptide hydrogel to repair a critical size skull bone defect in rat. While, at first, the presence of the M1 phenotype indicated inflammation, the authors showed how a transition towards an M2 phenotype could be achieved thanks to the incorporated glycopeptide. This glycopeptide had the ability to specifically bind to the CD206 receptor in macrophages, thereby inducing a phenotype transition. In turn, this change in macrophage polarization resulted in a considerably increase the proliferation of OBs and osteogenic differentiation of MSCs [25].

## Growth factors and the angiogenic-osteogenic coupling

Due to the prominent roles of osteogenesis and angiogenesis, the GFs utilized in bone tissue regeneration are mainly classified as osteogenic and angiogenic [26]. Osteogenesis, which is the process of bone formation, involves the well-coordinated differentiation and proliferation of osteogenic cells into OBs together with the production of the ECM [27]. The important mediators of osteogenesis include PDGF, TGF-β, FGF, IGF and BMPs and, therefore, they have been identified as osteogenic GFs. Amongst them, BMPs play a prominent role in bone healing [9]. The important role of BMPs in bone regeneration is highlighted by the fact that two of them (i.e., BMP-2 and BMP-7) have already been approved by the U.S. Food and Drug Administration (FDA) and are currently being used in the clinic for bone regeneration [28–30]. In turn, angiogenesis, which is the process by which new blood vessels are formed in the absence of pre-existing vascular components and starts with the activation of endothelial cells (ECs); is a crucial process to support proper bone remodeling [31]. Angiogenesis is also regulated by several mediators including IGF, FGF, PDGF and VEGF which are thus known as angiogenic factors. Importantly, several angiogenic GFs that are usually expressed during the early phases of blood vessel formation are also believed to play a crucial role in the formation of new bone in a process known as the angiogenic-osteogenic coupling. Amongst the different GFs involved in this coupling, VEGF, which has been shown to promote migration and proliferation of ECs, plays a critical role both as an angiogenic modulator for the formation of blood vessels but also in the process of bone formation. The crucial role that VEGF also plays in osteogenesis has been demonstrated in many studies. For example, Zhang et al. fabricated a VEGF/poly(lactic-*co*-glycolic acid) (PLGA)/fibrin composite which was used as a glue in a femoral neck fracture model using dogs [32]. The as-prepared VEGF-loaded glue was able to activate bone marrow (BM) stromal cells which resulted in the generation of new vessels. Interestingly, these activated BM stromal cells enhanced the release of osteogenic factors, such as BMP-2 which, in turn, significantly improved the quality and speed of fracture healing. PDGF is another GF that also participates in the angiogenic-osteogenic coupling. The angiogenic properties of PDGF arise from its ability to upregulate VEGF expression but it can also stimulate the growth of OBs by attracting pericytes to the injury site [33, 34]. As such, in a recent study, it was demonstrated that PDGF was able to enhance the osteogenic differentiation of BM-MSCs by inhibiting adipogenic differentiation by activating Ras-dependent extracellular signal-regulated kinase (ERK) 1/2 signaling pathways. In turn, regulation of phosphatidylinositol 3-kinase /protein kinase B (PI3K)/AKT and ERK1/2 signaling pathways enhanced angiogenesis and migration of human umbilical vein endothelial cells (HUVECs) [35]. Likewise, osteogenic GFs also have the ability of promoting angiogenesis as shown by several studies. For example, as early as in 2002, Martine et al. demonstrated how BMPs were able to stimulate angiogenesis by promoting the secretion of VEGF-A from OBs-like cells [36]. Another study showed that BMPs were able to induce p38 phosphorylation and to enhance the Smad signaling pathway to impact tube formation and EC proliferation [37, 38]. Actually, the effect of BMPs on bone regeneration has frequently been attributed to the stimulation of angiogenesis. However, the exact mechanism still has not been thoroughly described. Apart from the BMPs, other osteogenic GFs with the ability to affect angiogenesis include TGF-β or FGF-1 [39, 40]. For example, an in situ immunohistological analysis conducted by Kelpk et al*.* showed that, the incorporation of FGF-1 in a fibrin/HA-composite scaffold, promoted both angiogenesis and osteogenesis [40]. Specifically, it was shown how FGF-1 could increase the expression of osteogenic related markers such as osteopontin (OPN), osteocalcin (OCN) and alkaline phosphatase (ALP). While OPN is involved in adhesion, proliferation and migration of several bone related cells, OCN is the responsible gene ECM mineralization and ALP is a major regulator of bone mineralization [41, 42]. Moreover, FGF-1 may be a chemoattractant for inflammatory cells, delivering ECM components and diffusible mediators. These cells release cytokines (i.e., VEGF) that modulate the angiogenesis process. Therefore, these processes regulate specific biological cascade and promote mineralization, osteoblast cell maturation and vascularization [40].

### Drugs and small molecules

Apart from the GFs, several small molecular compounds and drugs can also act as osteogenic and angiogenic agents [43] (see Tables 1 and 2). For example, simvastatin, which is a cholesterol-lowering drug approved by the FDA, improves angiogenesis by upregulating the expression of VEGF in a dose dependent manner [44]. The angiogenesis related properties of simvastatin have already been verified in vivo [45]. According to another study, metformin, which is the gold standard drug for treating type 2 diabetes, can also be used as an osteogenic agent due to its ability to improve bone healing by stimulating the expression of collagen type I and OCN [46]. Apart from drugs, small molecules like α-tocopherol can also act as angiogenic factors via upregulating VEGF-R2 signaling pathway [47].

**Table 1:** A summary of angiogenic agents used in bone tissue engineering and their mechanism of action

Angiogenic agents		Mechanism of action	References
Chemical agent	OCP	Direct effect on cell migration and proliferation. Indirect effect by recruiting macrophages that induce secretion of cytokines such as VEGF	[182]
	Sr^2+^	Increases the level of pro-angiogenic factors (e.g., VEGF, MMP-2 and bFGF)	[72]
	Co^2+^	Activation of the HIF-1α pathway	[183]
	DMOG	Stimulation of the AKT/mTOR pathway in HUVECs	[123, 184]
	Mg^2+^	Promotes VEGF and endothelial nitric oxide secretion	[185]
	Fe^3+^	Increases the level of HIF-1α by decrease activation of prolyl hydroxylases	[186]
Drugs	Simvastatin	Enhances VEGF and HIF-1α expression	[44]
	PTHrP-2 (PTH derivative)	Increases expression of angiogenesis-related genes such as FGF and VEGF	[187]
	Deferoxamine	Activates the HIF-1α signal pathway and the overexpression of VEGF	[188, 189]
	Erythropoietin	Forms the erythropoietin and the erythropoietin receptor complex	[190]
Small molecules	CASIN and AMD3100	Enhances proliferation and migration of ECs	[191]
	ADTM	Enhances VEGF expression in vascular smooth muscle	[192]
	α-Tocopherol	Affects VEGF-R2 signaling pathway	[47]
	SalB	increase in VEGF and a major role on the Akt/mTOR/p70S6K signaling pathway	[167]
	Eu^3+^	Stimulates immune response of macrophages	[193]
	Salidroside	Increases VEGF and nitric oxide secretion	[194, 195]
	SP	Increases migration of BMSCs and VEGF expression	[196]
GFs	PDGF	Increases VEGF expression	[197]
	VEGF	Enhances ECs migration and increases vascular permeability	[198]
	FGF	Enhances ECs proliferation and differentiation	[199, 200]
Genes	ZEB1	Activates the PI3K and p38 pathways	[201]
	Foxc2	Activates PI3K and ERK	[202, 203]

OCP, octacalcium phosphate; Sr2+, strontium ion; VEGF, vascular endothelial growth factor; MMP-2, matrix metalloproteinase-2; bFGF, basic fibroblast growth factor; Co2+, Cobalt ion; HIF-1α, hypoxia-inducible factor 1α; DMOG, dimethyloxaloylglycine; HUVECs, human umbilical vein endothelial cells; Mg2+, magnesium ion; Fe3+, iron ion; PTHrP-2, parathyroid hormone derivative; ADTM, danshensu derivative (R)-(3,5,6-trimethylpyrazinyl) methyl-2-acetoxy-3-(3,4-diacetoxyphenyl) propanoate; SalB, salvianolic acid B; VEGF-R2, vascular endothelial growth factor receptor 2; Eu3+,europium ion; SP, substance P; GFs, growth factors; BMSCs, bone marrow stem cells; ECs, endothelial cells

**Table 2:** A summary of osteogenic agents used in bone tissue engineering and their mechanism of action

Osteogenic agents		Mechanism of action	References
Chemical agents	AuNPs	Activates the Wnt/β- catenin pathway	[204]
	Calcium carbonate	Activates ERK and p38 signaling pathways	[205]
	Oxy133	Activates the Hedgehog signalling pathway	[206]
	GO	Induces PGE2 secretion and BMP-2 expression	[207]
	Metformin	Increases expression BMP-2, Leptin, RUNX2 and activates AMPK pathway	[46]
	Ascorbic acid	Enhances ALP activity increasing collagen type 1 secretion	[208, 209]
	DEX	Activates TAZ, WNT/β-catenin and MKP-1	[208]
	UDCA	Reduces intracellular ROS and pro-inflammatory cytokines	[210]
Small molecules	SP	Induces autophagy by regulation of the AMPK / mTOR pathway	[211]
	Bergenin	Activated SIRT1 mRNA and protein level	[212]
	Probiotics	Increases the secretion of OSM by macrophages	[213]
	CORM	Enhances RUNX2, OPN, OCN and ALP activity	[214]
GFs	BMPs	Stimulate osteogenesis via Smads, MAPK pathways	[215, 216]
Genes	MiR-378	Activates Wnt signaling pathway	[217]
	MiR-21	Activates PTEN/PI3K/Akt/HIF-1α pathway	[218]
	MiR‐200c	Upregulates Wnt/β‐Catenin signaling	[219]
	MiR-675-5p	Activates HIF-1α and Wnt / β-catenin pathway	[220]
Others	LGR6	Activates the Wnt/ β‐Catenin signaling pathway	[221]
	MMP2 inhibitor	Activates the p38/mitogen-activated protein kinase pathway	[222]

Oxy133, semi-synthetic oxysterol; GO, graphene oxide; PGE2, prostaglandin E2; BMP, bone morphogenetic proteins; RUNX2, runt-related transcription factor 2; DEX, dexamethasone; UDCA, ursodeoxycholic acid; SP, substance P; OSM, oncostatin M; CORM, carbon monoxide releasing molecule; MiR-378, microRNA-378; LGR6, leucine-rich repeat containing G-protein-coupled receptor 6; MMP2 inhibitor, matrix metalloproteinase 2 inhibitor

## Specific properties of scaffold platforms

Scaffolds for BTE applications should mimic both the structure and properties of the natural ECM which is able to supply a suitable environment for cell adhesion, proliferation, osteogenic differentiation and vascularization while, at the same time, displaying appropriate mechanical features allowing for bone remodeling [48, 49]. Additionally, successful scaffold platforms should also allow for the incorporation of important active agents such as GFs, genes, small molecules or drugs [50]. Specifically, successful scaffold platforms should fulfil the following criteria: (i) being biocompatible, that is, providing the right conditions for normal cellular activity without provoking immunological response of the host; (ii) being biodegradable once the revitalization of the impaired bone has been completed and, importantly, the resulting degradation products should be nontoxic [51]. Additionally, the scaffolds should display (iii) appropriate mechanical strength which usually involves a Young's modulus ranging from 15 to 20 GPa and from 0.1 to 2 GPa for cortical and cancellous bone, respectively [52]. (iv) Being osteoinductive and osteoconductive in order to promote osteogenic differentiation and cellular proliferation is also a requirement and, lastly, displaying an architecture with a (v) interconnected porous micro- to- nano hierarchical structure is also essential. Actually, a large body of research has been devoted in the past years to study the effect that the pore size, porosity and pore interconnectivity of the scaffolds has on bone regeneration [53, 54]. This is due to the relevance that such features have in cell attachment, migration and vascular ingrowth [55]. While large pores facilitate the diffusion of oxygen and nutrients needed for vascular ingrowth, effective cell attachment is only possible if the pore size is small enough [52, 56]. However, the optimum pore size for BTE applications still remains to be identified as shown by the disparity found in different studies. For example, Boyan et al*.* found that bone formation was most favorable for scaffolds displaying pores in the size range of 200–400 μm [57]. Meanwhile, Hulbert et al*.* suggested that pore diameters of 100–350 μm were the optimal to promote bone in-growth [58]. In contrast, Correia et al*.* showed how, pore sizes of 400–600 μm, are beneficial for bone formation and vascularization [59]. Although pore size and porosity promote tissue ingrowth, it is important to note that scaffolds displaying high porosity with pores of large size will also display diminished mechanical strength which may compromise the scaffold’s integrity. Thus, a fine balance between pore size, porosity and sufficient mechanical strength needs to be attained.

Another physical parameter that can influence bone formation is the scaffolds nanotopography [60, 61]. The beneficial effects of introducing nanotopography onto the scaffolds are attributed to the increased surface area which results in enhanced cell adhesion, proliferation and cellular response [60–63]. For example, Jin et al*.* demonstrated how biomimetic scaffolds with surface nanotopography enhanced M2 macrophage polarization and IL-4 secretion as well as the osteogenic differentiation of MSCs by promoting their recruitment into the bone defect [24]. Specifically, the authors fabricated a hierarchical intrafibrillarly mineralized collagen (HIMC) using poly(acrylic acid)-calcium precursors, tropocollagen and HA [24]. The resulting scaffold platform displayed a bone-like staggered nanointerface with immunomodulatory properties. As such, the study showed how the macrophages were able to adhere to HIMC’s collagen fibers which, in turn, promoted the macrophages polarization into the M2 phenotype, enhanced IL-4 release and improved MSC osteogenesis.

Integration of all the aforementioned properties within a specific scaffold platform is, obviously, not an easy task. For that, a wide range of technologies have been developed and will be discussed in the next section.

## Fabrication techniques

Scaffold fabrication techniques can be classified into conventional and modern techniques.

### Conventional methods

The conventional methods include particle leaching and solvent casting, gas foaming and thermal-induced phase separation (TIPS) [64].

Particle leaching and the solvent casting technique are relatively simple, inexpensive techniques where a polymer is dissolved in an organic solvent and mixed with a water-soluble porogen (i.e., a salt such as sodium chloride or sodium citrate). The resulting mixture is casted into a mold and, following evaporation or freeze-drying of the solvent, the polymer/porogen composite is leached into water [65]. With such an approach, both the pore size and porosity can be easily controlled by adjusting the salt and polymer ratio (50–90%) [64, 66]. However, with this method, the remaining toxic solvent can cause denaturation of incorporated fragile biomolecules [64].

With the freeze-drying technique, a water-based polymer solution is frozen which results in polymer aggregation within the interstitial spaces around the ice crystals. Upon applying vacuum, dry interconnected porous scaffolds are obtained by complete solvent sublimation [67]. In this context, an important parameter that needs to be controlled is the freezing direction since it has a huge impact on the pore morphology of the resulting scaffolds. With freeze-drying, porogens are not needed however, limitations of this technique include the resulting irregular porosity and the small size of the pores.

With the gas foaming method pores are created by gas expansion and carbon dioxide, which is a low-toxic and non-flammable gas, is used as the porogen [65]. This method renders scaffolds with sponge-like structure since an 85% porosity can be achieved. Additionally, this method can be used at ambient temperature and with aqueous solvents which can protect the activity of the encapsulated active compounds [64, 68]. The main disadvantage of this method is that the resulting scaffolds may have a closed pore or a solid polymeric skin structure.

The TIPS technique relies in dissolving a polymer in a solvent at a high temperature and, upon cooling down the solution, a phase separation takes place and a microporous structure arises following solvent removal [65]. The properties of the resulting scaffolds (i.e., pore size, morphology, bioactivity and degradation rate) can be controlled by adjusting the polymer concentration and the volume of the secondary phase fraction. A drawback of this technique is the rather small pore size (i.e., 10–100 µm) which is suboptimal to promote bone-tissue growth.

### Modern methods

The more recent methods developed for scaffold fabrication include electrospinning, additive manufacturing (AM) techniques and photolithography. Unlike conventional methods, these fabrication techniques can provide scaffolds with structures that better resemble the architecture of the ECM of the bone tissue. Also, they enable the production of scaffolds with enhanced mechanical stability.

#### Electrospinning

Electrospinning is a technique where a high electric field is used to produce micro- or nanofibers by reduction of surface tension within the polymer fluids. The melted or in solution polymers are injected with an electrical potential to create a charge imbalance which results on the stable and steady deposition of electrospun fibers onto any substrate [65]. The formation of nanofibrous matrices by electrospinning has attracted a lot of attention for BTE applications since it is a simple process where a wide range of polymers can be used. Additionally, the resulting scaffolds display a high surface area to volume ratio thus mimicking the natural ECM [69]. Additionally, active compounds can also be incorporated within the fibers. As such, on a recent example, simvastatin and dexamethasone (DEX) as pro-angiogenic and osteoinductive factors, respectively, were incorporated into a PCL-collagen fibrous scaffold fabricated by electrospinning [70]. The results showed that the functionality of both drugs was preserved during the electrospinning process as shown by increased cell proliferation and enhanced osteogenic differentiation up to 21 days. The angioinductive nature of the dual drug-loaded fibers was demonstrated by enhanced tube formation and a twofold higher angiogenic score upon culturing primary HUVEC cells.

Although the nature of the solvent and the high voltages applied during the electrospinning process may denature any encapsulated biomolecule, there are several studies demonstrating that the local delivery of both angiogenic and osteogenic factors through electrospun nanofibrous scaffolds is also possible. For instance, Shruthy et al*.* incorporated FGF-2 and BMP-2 and VEGF within a scaffold of silica coated nano HA-gelatin reinforced with electrospun poly(*L*-lactic acid) (PLLA) yarns [71]. The results showed how, the GF-loaded scaffold, was able to promote vascularization and bone regeneration in critical sized calvarial defect. Other active compounds different than GFs can also be incorporated within the electrospun fibers. For example, Ye et al*.* embedded strontium (Sr^2+^)-doped calcium phosphate (Ca-P) NPs within a PCL/CHI matrix also by electrospinning [72]. While Sr^2+^ was incorporated due to its important role in bone metabolism, Ca-P was chosen since it has a chemical composition similar to the inorganic components of native bone. The resulting Sr^2+^-Ca-P NPs/PCL/CHI fibers were able to promote both osteogenesis and angiogenesis, indicating a potential synergism between the Sr^2+^ and the Ca-P from the fibers.

However, scaffolds fabricated by traditional electrospinning methods display a 3D structure with low porosity. Thus, a novel extension of electrospinning, which is the so-called coaxial electrospinning has been introduced for the fabrication of unique core–shell nanofibers. During coaxial electrospinning, two different solutions are ejected through a coaxial nozzle with a core–shell structure. Importantly, with this modality, fragile biomolecules can be incorporated into the core solution thus being protected from exposure to harsh solvents [73]. As a recent example, poly(lactic acid) (PLA)-based scaffolds encapsulating BMP-2 and tauroursodeoxycholic acid (TUDCA), which is a vascular inducer, have been reported [74]. In particular, a TUDCA/PLA solution was coaxially electrospun as the sheath fiber while a PLA/BMP-2 solution was the core fluid [74]. With such an approach, TUDCA showed a burst release from the PLA nanofibers followed by a sustained release pattern for BMP-2. The results showed that such a release pattern was beneficial for long-term bone healing. Despite the many advantages, coaxial electrospinning displays also some drawbacks such as complex setup, clogging of the nozzle and low scalability potential since the repulsive interaction between adjacent liquid jets leads to a low production rate [75, 76].

An interesting modality of electrospinning is the so-called multi-source multi-power (MSMP) electrospinning (MSMP-ES) approach which was developed for manufacturing multicomponent fibrous scaffolds since it allows to independently modify processing parameters [77]. With MSMP it is possible to electrospun multi-component polymeric solutions [78, 79]. This is advantageous because, in some solvent systems, some polymers cannot be electrospun, but the addition of another polymer might result in an electro spinnable solution [77]. As an example, Wang et al. employed MSMP-ES to fabricate a multicomponent nanofibrous scaffolds where the PLGA/poly(ethylene glycol) (PEG) nanofibers where loaded with bioactive Ca-P NPs, recombinant human (rh) VEGF and rhBMP-2 [80]. The results showed improved bone regeneration and angiogenesis in a cranial defect mouse model. Limitations of scaffolds prepared by MSMP-ES include a restricted loading level of GFs and an inadequate compressive strength [81].

#### Additive manufacturing technique

The additive manufacturing (AM) techniques involve a variety of methods where the scaffolds are fabricated by adding and processing the materials in a layer-by-layer fashion making use of commercial computer-aided design (CAD) tools. AM techniques render scaffolds with precisely predefined internal and external architectures which makes them very well suited for BTE applications. The most widely used AM techniques include 3D printing, fused deposition modeling (FDM), and selective laser sintering (SLS) [67].

##### 3D printing

3D printing is a fabrication technique where a wide range of materials (i.e., ceramics, plastics, metals, liquids or even living cells) are used as bioink to produce 3D constructs by adding them successively in a layer-by-layer fashion. The properties of the bioink which will have an important effect on the resulting scaffold include viscosity, gelation and cross-linking which will, in turn, affect cell attachment, viability and proliferation. The different 3D printing methods developed to date include:include extrusion-based bioprinting, inkjet bioprinting and laser-assisted bioprinting.

During extrusion bioprinting, an extrusion-based printing system is used to extrude the biomaterials through a micronozzle without any heating process. With this technique, the 3D scaffolds are created by the deposition of biomaterials mixed with cells in the XY plane of the stationary print bed followed by Z-axis in a layer-by-layer fashion. While this method renders porous scaffolds that will promote cell proliferation, the viability of the printed cells is affected and, furthermore, cell distortion takes place due to the applied shear stress or pressure [67]. In an attempt to promote enhanced cell viability, Cidonio et al. incorporated synthetic nanoclay laponite (LPN) into a GelMA bioink containing also VEGF [82]. To create the scaffolds, the bioink was extruded using a computer-assisted syringe dispenser at 25 °C and 4–10 layer scaffolds were deposited and exposed to visible light for 5 min. Their results showed how the incorporation of LPN into the GelMA bioink improved cell viability, stimulated osteogenic differentiation while also improving the physical integrity and the interconnected porosity of the scaffolds.

**Fig. 2:**
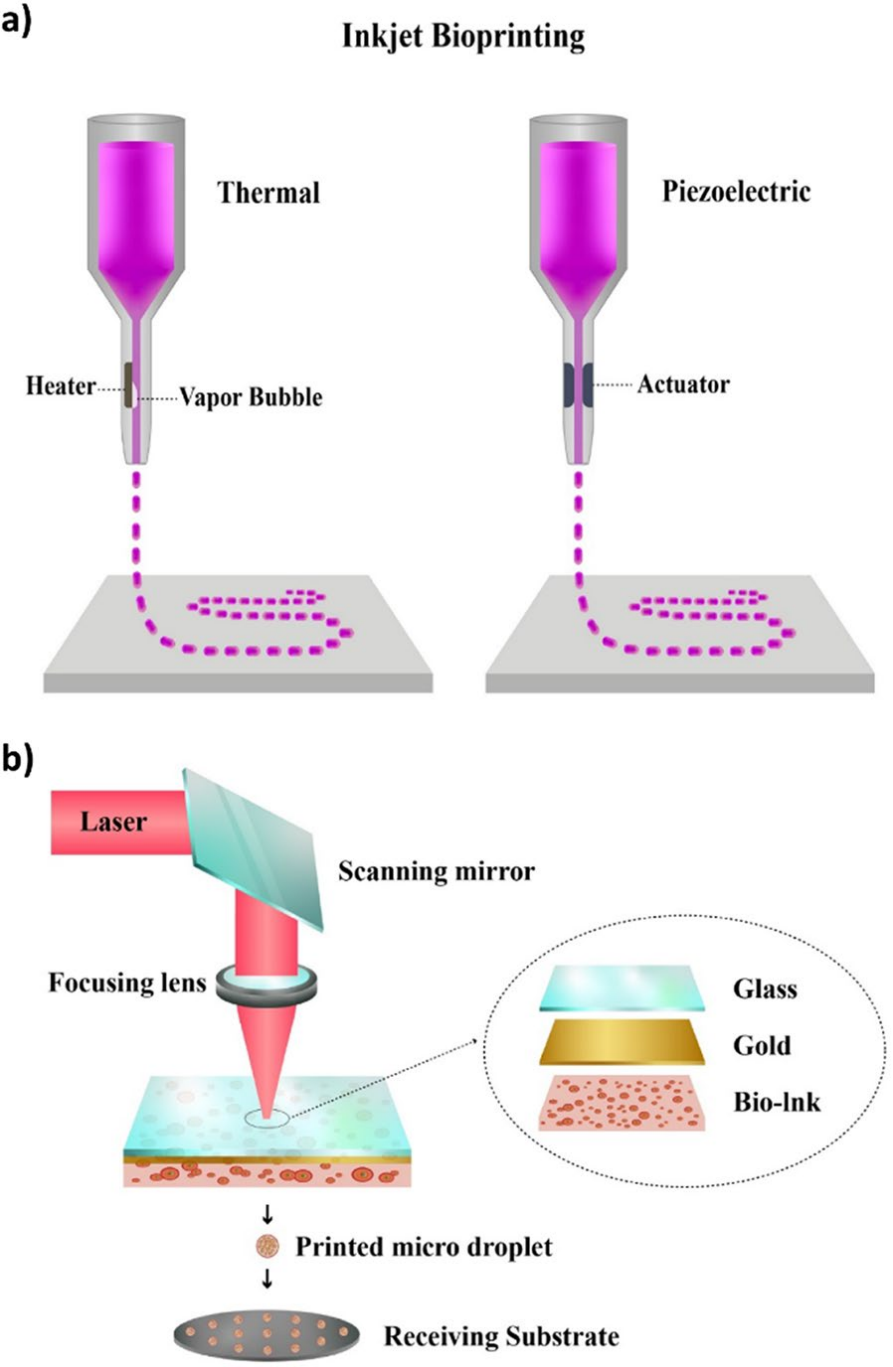
**a** Schematic of inkjet bioprinting: a pulse thermal or piezoelectric stimulation causes tiny droplets of bioink to be released from the nozzle. **b** Laser-assisted bioprinting: includes a laser pulse, the ribbon (a transparent glass covered with a thin layer of laser absorbing metal and bioink) and a receiving substrate

Inkjet bioprinting also known as droplet bioprinting, facilitates the fabrication of a tissue construct via ejecting a cell-laden bioink from a nozzle [83, 84]. The printer head uses piezoelectric or thermal force to squeeze the biomaterials out of the nozzle and produce a droplet of a controllable size which will be deposited onto a substrate (Fig. 2a). The benefits of this method include low cost, fast printing speed as well as easy access [84]. An example of a scaffold fabricated by inkjet bioprinting is the work by Gao et al. [85]. Specifically, the authors printed PEG dimethacrylate scaffolds enriched with osteoinductive HA NPs in combination with human MSCs. Following 21 days of incubation, enhanced collagen production, ALP activity and cell viability were observed when the MSCs where embedded in the bioink as compared to when they were manually pipetted. The main limitation of this method is that only low viscosity bioinks and with low concentrations of cells can be used. To overcome this limitation, a novel direct-volumetric drop-on-demand strategy that enables dispensing bioinks with a high concentration of cells and highly viscose biomaterials has been reported [86].

Laser-assisted bioprinting is a nozzle-free technique where a laser beam is used to accurately deposit the biomaterials onto a substrate (Fig. 2b) [67, 87, 88]. This type of bioprinting is a non-contact procedure that offers a high degree of precision and resolution which makes it suitable for bioprinting DNA, cell arrays and micropatterned peptides. Keriquel et al. used laser-assisted bioprinting technology to fabricate a scaffold composed by collagen and nano-HA NPs and MSCs. Their results demonstrated improved bone formation and osteoconductivity on a calvarial defect site after 2 months as compared to scaffolds containing only collagen and nano-HA NPs [89]. Roskies et al. fabricated scaffolds where polyetherketoneketone (PEKK) was used in combination with adipose-derived (AD) SCs [90]. They found that the laser-assisted bioprinted PEKK scaffolds showed desirable bioactivity, biomechanical strength and biocompatibility as well as improved bone implant interface using a rabbit model. However, this method has also several limitations such as the complexity associated with the laser printing control system and the high cost of the equipment [91]. Additionally, exposing the cells to a laser source can also decrease their viability [92].

##### Fused deposition modeling

**Fig. 3:**
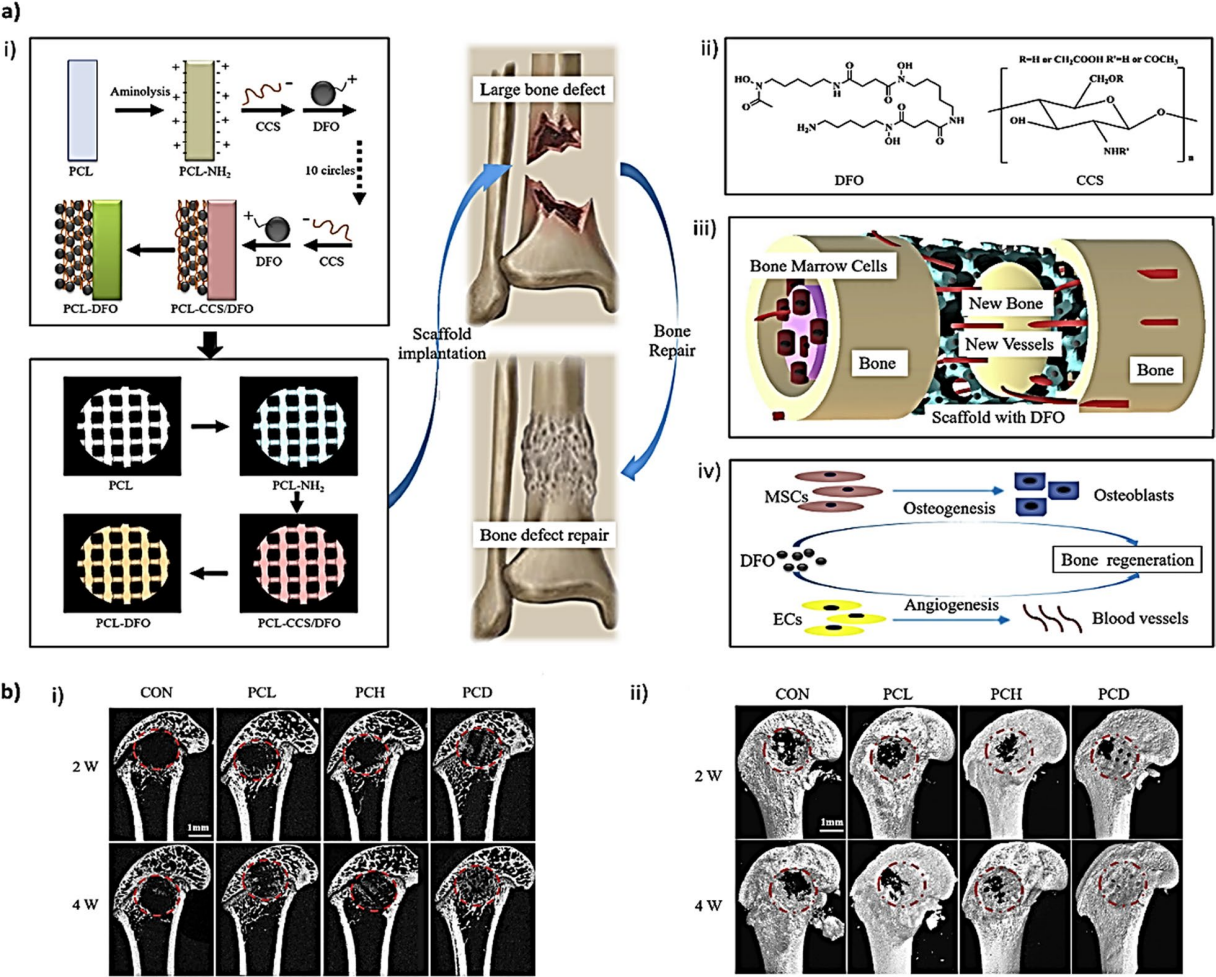
**a** Schematic illustration of bridging deferoxamine (DFO) on the surface of 3D printed polycaprolactone (PCL) scaffold and its biological function for bone regeneration. i) Upper panel: Diagram showing the preparation process of PCL-DFO scaffolds including surface aminolysis and layer-by-layer assembly with oppositely charged carboxymethyl chitosan (CCS). Lower panel: Four scaffolds were used in animal study including the pure PCL, their intermediate product PCL-NH_2_, and the final product PCL-DFO. ii) The chemical molecular structure of DFO (left) and CCS (right). iii) Schematic illustration showing the effect of PCL-DFO scaffold on angiogenesis and osteogenesis at the bone defect site. iv) The cellular mechanism of promoting bone regeneration by DFO in mesenchymal stem cells (MSCs) and in vascular endothelia cells (ECs). **b** Micro-CT analysis of the effect of scaffolds on bone repair in vivo. The identified scaffolds (polycaprolactone (PCL), aminated PCL (PCH) and deferoxamine-loaded PCL (PCD) were implanted into the femur defect of rats, and rats without scaffolds were used as control (CON). **c** Representative two-dimensional micro-CT images (i) and three-dimensional reconstructed micro-CT images (ii) showing the effect of different scaffolds on the new bone tissue formation inside the defect site. The bone defect area was circled by dot line (red). Reproduced from [90] with permission

Fused deposition modeling (FDM) is a solvent-free fabrication method where a filament of the desired material is fed into a vessel, melted with heat, then extruded through a nozzle and deposited in a layer-by-layer fashion to build a scaffold. Yan et al. used FDM to produce scaffolds composed by PCL, 6-hexanediamine and coated by carboxymethyl CHI and deferoxamine as the angiogenic agent (Fig. 3). The final scaffold platform was used in a rat large bone defect model and the results showed enhanced vascularity regeneration leading to more bone formation and osseointegration [93]. Limitations of such a technique include the high temperatures needed to form and subsequently melt the filament [94].

##### Selective laser sintering

With selective laser sintering (SLS) the temperature of a material (e.g., plastic, metal, ceramic, or glass powder) is increased making use of a high-power laser beam to fuse the powder in a layer-by-layer form without melting it thus attaining a 3D construct. In this regard, Duan et al. also utilized SLS to manufacture nanocomposite scaffolds made of Ca-P/poly(hydroxybutyrate-*co*-hydroxy valerate) (PHBV) which was subsequently grafted with heparin for rhBMP-2 conjugation. Such an approach resulted in a sustained release of rhBMP-2. According to their findings, the utilization of SLS has the potential to yield porous and complex structures. Additionally, the results of the study demonstrated that such a sustained release pattern of rhBMP-2 could greatly improve osteogenesis during 21 days [95]. In another investigation, SLS was employed to fabricate scaffolds composed of borate BG (BBG) and PCL with the aim of directing the regeneration process for critical sized bone defects (CSBD). The findings of this study revealed that BBG/PCL scaffolds display desirable degradation properties, suitable mechanical characteristics and also possess the ability to stimulate osteogenesis and osseointegration. Therefore, SLS exhibited great potential for manufacturing BBG/PCL composite scaffolds with customized size, shape and internal porous structures for directed regeneration of CSBD [96].

Although this method has notable advantages, certain challenges still need to be addressed. The achievable resolution is reliant on the laser beam diameter, making it an expensive technique. Moreover, the high temperature necessary for the process prohibits the direct incorporation of viable cells and biomaterials into the scaffold. Another drawback is the extended production time required in comparison to alternative methods [97].

#### Photolithography

Photolithography is a top-down approach that involves transferring a geometric pattern from a photomask to a light-sensitive photoresist on a substrate. The transfer of the pattern to the photoresist is usually done by exposing UV light through the photomask. Photolithography can be used to pattern different biomaterials including cells or proteins, however, the maintenance and cleanliness required by this instrument is a drawback of this method.

**Fig. 4:**
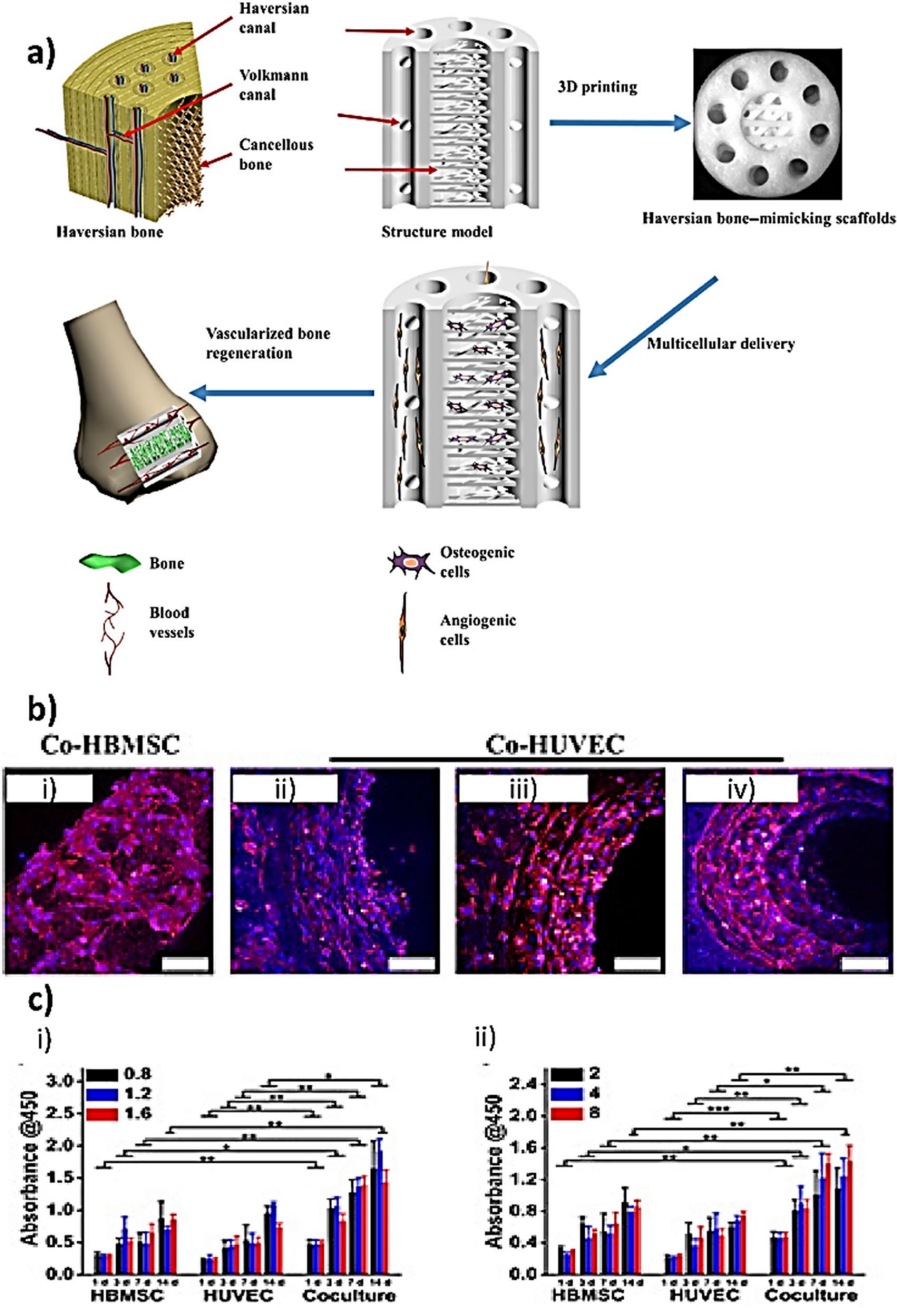
**a** Angiogenic and osteogenic cells can be delivered through Haversian bone–mimicking scaffolds 3D printed with Volkmann canals, Haversian canals and cancellous bone structure. In the scaffolds' cancellous bone structure, osteogenic cells were implanted while the angiogenic cells were seeded on the Haversian canals. The multicellular delivery of Haversian bone–mimicking structure contributed to new bone and vessel formation. **b** (i to iv) Confocal laser scanning microscopy images of human bone marrow stem cells (HBMSCs) seeded on the cancellous bone structure and human umbilical vein endothelial cells (HUVECs) seeded on the Haversian canal with varying diameters. **c** Proliferative activity of HUVEC, HBMSC, and cocultured HBMSC-HUVEC seeding on scaffolds with varying (i) diameters and (ii) numbers of Haversian canals after culturing for 14 days. Reproduced from [92] with permission

A relevant photolithographic technique is stereolithography (SLA) which is a fast method with excellent resolution and improved cell viability. In SLA, a liquid-based biomaterial containing a photoinitiator molecule is continuously exposed to a laser beam (UV or visible light). The incoming light and the photoinitiator trigger a local polymerization reaction, which results in curing only in the exposed portions. This leads to the deposition of a first layer, followed by the application of a fresh film, which is also irradiated and cured. This process renders a solidified photosensitive biomaterial in a layer-by-layer fashion. SLA was used by Zhang et al. to fabricate a haversian bone–mimicking scaffold for multicellular delivery to enhance bone regeneration (Fig. 4). The as-prepared scaffolds were able to promote angiogenesis, osteogenesis and neurogenic differentiation in vitro and accelerated bone formation in vivo [98].

## Scaffold-based strategies for simultaneous angiogenesis and osteogenesis

To develop functional scaffold platforms in bone tissue engineering, the administered cells should be guided in a controlled manner using a combination of mechanical signals, conducting genes and bioactive agents [99]. In this part, we focus on NP-incorporating scaffolds that enhance the retention time of the biologicals in the scaffold platform and reduce the systemic adverse reactions related to their high dose and burst release. Another important category is the gene-eluting scaffolds which facilitate high transfection of the incorporated cells with genes inducing angiogenic and osteogenic properties. Also, we introduce multifunctional scaffolds that enable the loading of various cell types, GFs or genes to provide ECM mimicking structures.

### NPs-incorporating scaffolds

In order to incorporate bioactive agents with the ability to enhance osteogenesis and angiogenesis, scaffolds incorporating NPs have been developed. Three main types of NPs have been considered: nanoparticulate carriers loaded with bioactive compounds, inorganic NPs with intrinsic osteogenic properties and biologically derived exosomes.

#### Bioactive agents-loaded NPs

GFs, drugs and other small molecules have been considered as bioactive agents to induce both angiogenesis and osteogenesis. In order to enhance their retention time within the scaffold platform and reduce the systemic adverse reactions related to a potential high dose and burst release, these compounds have been encapsulated within a wide range of nanoparticulate carriers. This is of particular relevance for GFs which have an extremely short half-life in circulation (usually of only a couple of minutes) due to their fast in vivo degradation [100]. In this context, Wei et al. employed PLGA NPs to entrap BMP-7 which were subsequently loaded into PLA-based scaffolds. Interestingly, the in vitro release kinetics showed that, by means of the PLGA NPs, a sustained release of BMP-7 for up to six weeks could be attained [101]. Besides, when BMP-7 was incorporated into the scaffold without the NPs, the results indicated loss of biological function and the subsequent failure in inducing bone formation. Similarly, BMP-2 has also been incorporated into a PLA-based scaffold by using a liposomal formulation [13]. The sustained release of the GF over a 21-day period resulted on the osteogenic differentiation of pre-seeded MSCs.

Instead of GFs, drugs such as DEX have also been employed as osteogenic inducers due to their long-lasting activity, low molecular weight and high potency [102]. To achieve a sustained release, Chen et al. prepared DEX-loaded biphasic Ca-P-based NPs which were subsequently hybridized with collagen [103]. The resulting composite scaffold was able to induce both osteogenesis and angiogenesis. The latter was attributed to the scaffold’s microgroove network which promoted the alignment of HUVECs into tubular structures. In another investigation, mesoporous silica NPs (MSNPs) were used as the carrier for dual angiogenic-osteogenic factor delivery [104]. The MS-NP were first modified with CHI to achieve improved biocompatibility, and subsequently loaded with DEX and the QK peptide as the osteogenic and angiogenic factors, respectively. While DEX was incorporated within the mesoporous channels of the MS NPs, the QK peptide was immobilized on the outer surface. The in vitro results showed how, by using MSNPs, a fast release of QK and a sustained DEX release could be achieved which resulted in the induction of both osteogenesis and angiogenesis. This enhanced osteogenesis and angiogenesis was also attributed to the release of silicon ions (Si^4+^) from the MSNPs [104].

Small molecules as bioactive agents have also been considered. For example, Shang et al. fabricated PLGA nanofibers incorporating both silicate nanoplatelets and dimethyloxalylglycine (DMOG) as an angiogenic small molecule [105]. While DMOG is a cell permeable agent and a prolyl hydroxylase inhibitor which suppresses the catabolism of hypoxia-inducible factor 1 (HIF-1) leading to upregulation of angiogenesis, the silicate nanoplatelets display osteogenic properties. Specifically, silicate nanoplatelets display a dual charge distribution due to a negatively charged silica surface with positively charged edges due to the substitution of magnesium ions (Mg^2+^) by lithium ions (Li^+^). The presence of Mg^2+^ in this structure promotes cellular adhesion, enhancement of VEGF expression in undifferentiated MSCs together with osteogenesis induction. The results of the study revealed that angiogenesis and osteogenesis were significantly improved through recruitment of CD90^+^/CD34^−^ stromal cells which also led to enhanced bone tissue regeneration.

All in all, these studies highlight the tremendous benefit of using NPs to incorporate several active compounds to promote both osteogenesis and angiogenesis. Other examples can be found in Table 3 which shows a summary of different kinds of NPs encapsulating osteogenic/angiogenic agents (i.e., GFs, drugs and small molecule compounds) for BTE applications. However, despite the potential, it still remains as a challenge to achieve a high drug loading, a narrow size distribution and appropriate drug release kinetics. Biocompatibility and the degradation profile together with the potential for scaling-up of the manufacturing process, are other aspects that should also be considered [106]. However, we believe that the challenges associated with the use of NPs should not overshadow their tremendous potential. Instead, further studies must be conducted aimed specifically at investigating osteogenic and angiogenic NPs to improve their efficacy before they can be translated into the clinic.

**Table 3:** Various NP encapsulating osteogenic/angiogenic agents for bone regeneration

Angiogenic/osteogenic factor	NP type	Size (nm)	Details	References
EGF	Liposome	100	Sustained and continuous EGF release from the liposomes led to enhanced bone regeneration	[223]
Deferoxamine	TNTs	70	Sustained release was achieved which promoted osteogenic and angiogenic differentiation. The TNTs were coated with CHI and gelatin layers to enhance their biocompatibility	[189]
Cu^2+^	MSN	100	Sustained release of Cu^2+^ and Si^4+^ which enhanced both osteogenesis and angiogenesis	[224]
Paclitaxel, Deferoxamine, BMP-2	Liposome-modified hydrogel	220–450	Sustained release of paclitaxel, deferoxamine and BMP-2 from liposomes that had been embedded into a gelatin scaffold resulted in osteogenesis and early vascularization	[225]
DEX and QK peptide	MS NPs	170	A fast release of QK peptide (which had been incorporated into the MS NPs surface) and a DEX sustained release (which had been loaded into the MS NPs pores) together with the release of Si^4+^ enhanced both angiogenesis and osteogenesis	[104]
–	AuNPs incorporated into MS NP	80–110	Au NPs were able to modulate the secretion of osteogenic cytokines by macrophages. The continuous release of Si^4+^ ions promoted angiogenesis and osteogenesis	[226]
Sr^2+^	Bioactive glass NPs	75	Sr-doped bio glass NPs enhanced osteoblast activity and also stimulated osteoblasts to release angiogenesis-related cytokines for early vascularization	[227]
Eu^2+^	MS NPs	280–300	MS-NP modulated the macrophages inflammatory response while also promoting angiogenic/osteogenic differentiation by enhancing angiogenic (i.e., VEGFR1/2, CD31, MMP9) and osteogenic genes (i.e., ALP, OPN and OCN)	[193]

EGF, epidermal growth factor; TNTs, titania nanotubes; CHI, chitosan; MSN, mesoporous silica nanospheres; BMP, bone morphogenetic proteins; MSNPs, mesoporous silica nanoparticles; Au NPs, gold nanoparticle; Sr^2+^, strontium ion; Eu^2+^, europium ion; MMP-9, matrix metalloproteinase-9; CD 31, platelet endothelial cell adhesion molecule-1; VEGFR1/R2, vascular endothelial growth factor receptor 1/receptor 2; ALP, alkaline phosphatase; OPN, osteopontin; OCN, osteocalcin; DEX, dexamethasone

#### Inorganic NPs

Inorganic NPs such as nano-HA crystals or silica (SiO_2_) NPs have also been incorporated within scaffolds due to their osteogenic properties [107]. This is due to their ability to mimic the bone ECM which is constituted by collagen nanofibrils and nano-HA as the organic and inorganic components, respectively. Apart from being able to mimic the bone matrix at the nanoscale level and to display essential topographical features, such inorganic NPs have the ability to provide relevant minerals and other factors to enhance osteogenic differentiation, proliferation and cell migration [108].

As such, MS NPs have received a great deal of attention for BTE applications not only for their excellent biocompatibility, large surface areas and pore-volume ratios which makes them excellent candidates for the incorporation of active compounds; but also for their intrinsic osteogenic and angiogenic properties which are attributed to the Si^4+^ release from the NPs [109]. Specifically, a recent report has shown the ability of Si^4+^ to inhibit the Nuclear factor-kappaB (NF-κB)signaling pathway resulting in increased ALP activity, OCN expression, calcium deposition and vessel formation [110]. Thus, it has also been reported that NF-κB inhibitors, in addition to being anti-inflammatory and having the ability to suppress osteoclast formation, also enhance bone formation [111].

Apart from their intrinsic bioactivity, the incorporation of inorganic NPs has received significant attention to render scaffolds with sufficient mechanical strength which can be used for load bearing applications [112]. For example, the addition of both BG-based NPs and HA NPs has shown to enhance the compressive moduli of the resulting scaffolds [85]. Specifically, Gao et al. incorporated both types of NPs into a PEG dimethyl acrylate layer-by-layer 3D printed scaffold [85]. Their results showed how the HA NPs were better in enhancing collagen production, ALP activity and cell viability of pre-seeded hMSCs after 21 days of culturing period as compared to the BG NPs [85]. Importantly, both types of NPs were able to significantly increase the compressive modulus of the resulting scaffold.

#### Exosomes

Exosomes are a type of naturally-occurring vesicles excreted by various cell types which play critical roles in cell–cell communication [113]. They are constituted by lipid bilayers resulting in vesicles of about 30–200 nm in size incorporating several types of functional biomolecules such as micro RNAs (miRNAs), mRNAs or proteins [114, 115]. By triggering gene transcription, exosomes modulate cell function and play critical roles in different physiological processes [116].

The intrinsic ability of exosomes to regenerate bone tissue has been demonstrated in several settings. For example, Lou et al. have demonstrated that exosomes have the ability to treat osteoporosis [117]. By delivering aptamer-decorated exosomes to BM-derived SCs, they were able to enhance the bone mass in ovariectomized mice and to promote bone healing in a femur fracture mouse model. The angiogenic properties of exosomes have also been demonstrated. As an example, exosomes obtained from human dental pulp were able to induce angiogenic differentiation of vascular ECs [118]. On a different study, Zou et al*.* obtained exosomes from human CD34^+^ SCs that had been previously transfected with mi-R26a which is a potent osteogenesis inducer. The resulting exosomes, which were also CD34^+^, could efficiently inhibit femoral head damage caused by glucocorticoids by promoting concurrent osteogenesis and angiogenesis [119]. The angiogenic properties of exosomes have been evaluated in the context of distraction osteogenesis procedure. Distraction osteogenesis is the first-line therapy option for long bone defects caused by surgical resection or trauma, particularly in situations of postsurgical problems and infections. To promote angiogenesis, Jia et al. used exosomes derived from endothelial progenitor cells (EPCs) which were locally injected into distraction gaps [120]. The results showed how the injected exosomes were able to accelerate bone regeneration through stimulating angiogenesis.

Considering these outstanding osteogenic and angiogenic properties, exosomes have also been employed to create functional scaffolds. For example, Zhang et al*.* made use of exosomes from human-induced pluripotent SCs (hiPSCs)-derived MSCs to incorporate them into a tricalcium phosphate (TCP)-based scaffold which was subsequently used for the regeneration of rat critical sized calvarial bone defects [121]. The authors analyzed the effect of the exosome-containing scaffold on the gene expression profile of pre-seeded human-BM (hBM)-derived MSCs (hBM-MSCs). The results revealed that activation of PI3K/Akt signaling pathway could be the key mechanism for the observed osteogenesis enhancement. Specifically, the authors found that PDGF-α, FGF receptor 1, collagen type α I and II and BCL2-like 1 were significantly upregulated [121]. On a very recent study, exosomes derived from human AD-SCs (hAD-SCs) were embedded within a PLGA/Mg^2+^-gallic acid (GA)-organic framework nanofibrous scaffold prepared by electrospinning [122]. Both the in vivo and in vitro results indicated a sustained release of Mg^2+^, GA and exosomes from the scaffolds for up to 10 days. Importantly, the scaffold was able to induce both osteogenesis and angiogenesis. It was hypothesized that the slowly released exosomes were phagocytosed by co-cultured cells thus enhancing their anti-inflammatory properties and increasing the expression of VEGF, OCN, ALP and runt-related transcription factor 2 (RUNX2) [122].

In order to enrich the exosomes in terms of angiogenic or osteogenic activity, another strategy used in several studies relies in inducing the expression of GFs within the cells from which the exosomes are going to be derived. For example, Liang et al*.* pretreated hMSCs with DMOG to promote the secretion of angiogenic GFs [123]. The resulting DMOG-hMSCs derived exosomes were incorporated into HA-based scaffolds and evaluated by making use of a critical sized calvarial defect rat model. The enhanced bone formation was attributed to the exosomes ability to activate AKT/mTOR pathway to stimulate the angiogenesis of ECs [123]. Since several reports are suggesting that noggin expression in MSCs acts as a natural BMP antagonist, on another study exosomes were derived from hMSCs with downregulated noggin expression [124]. The extracted exosomes were then incorporated within a CHI-based scaffold which was subsequently used in non-healing calvarial defects mice model. The intervention resulted in robust bone regeneration.

As highlighted by these selected examples, great steps have been taken towards modifying exosomes in order to create a novel type of functional NP with potential in BTE. However, there are still many challenges that need to be overcome. Specifically, further research should be focused on rendering cell types with a strong ability to secrete angiogenesis/osteogenesis-promoting exosomes to obtain sufficient amounts for biomedical applications.

### Scaffolds for gene delivery

Another promising strategy to enhance bone regeneration is the incorporation of therapeutic nucleic acids within the scaffolds. In contrast to the direct delivery of GFs, gene delivery is considered to be more cost-effective since it provides physiological amounts of the target protein as compared to the administration of active GFs at the site of action [125]. Gene delivery is also regarded as a less immunogenic approach [126]. There are several types of nucleic acids used to create gene-activated scaffolds for BTE applications: plasmid DNA (pDNA), small interfering RNA (siRNA) and miRNA [127].

**Fig. 5:**
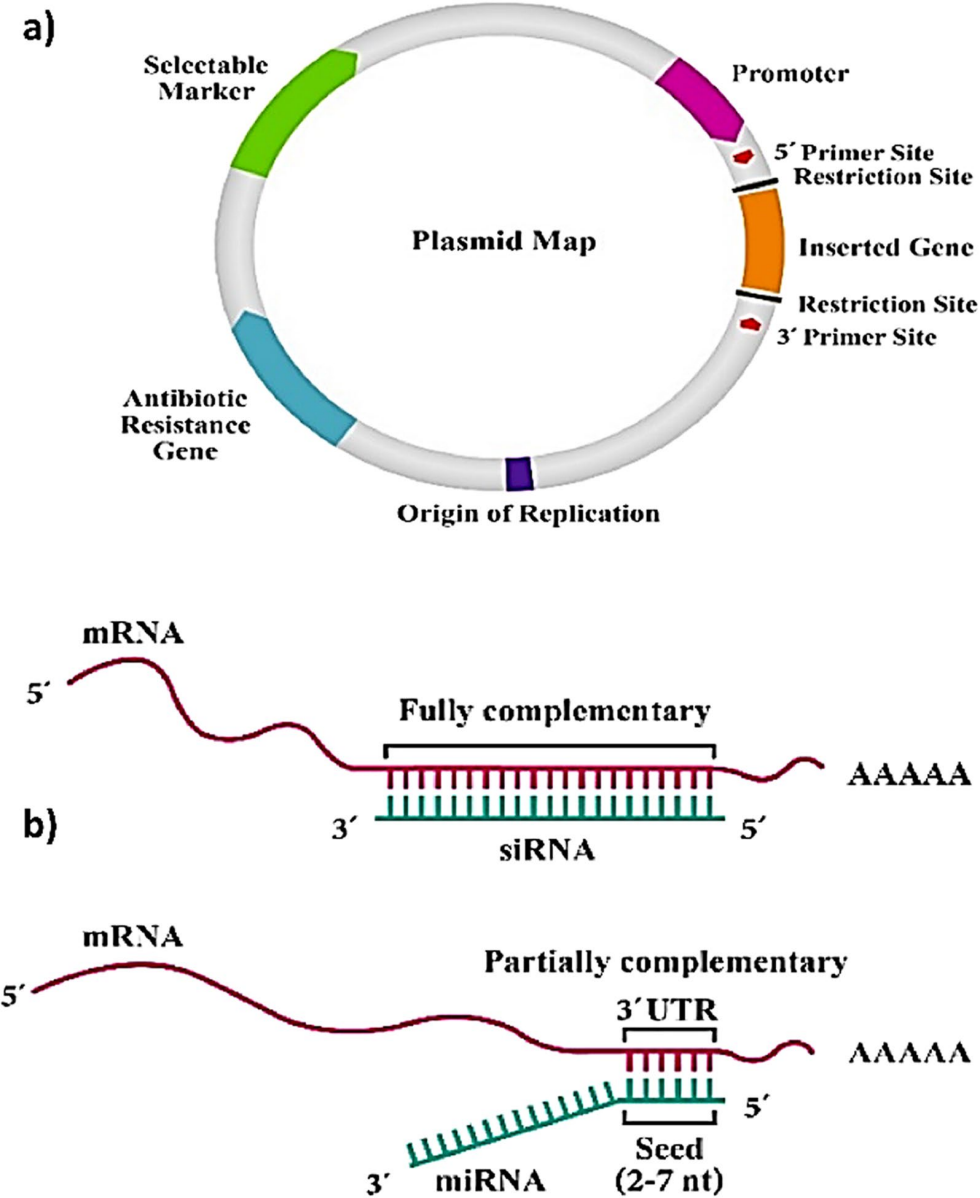
**a** Schematic illustration of a plasmid. **b** Target recognition by siRNA and miRNA

**Fig. 6:**
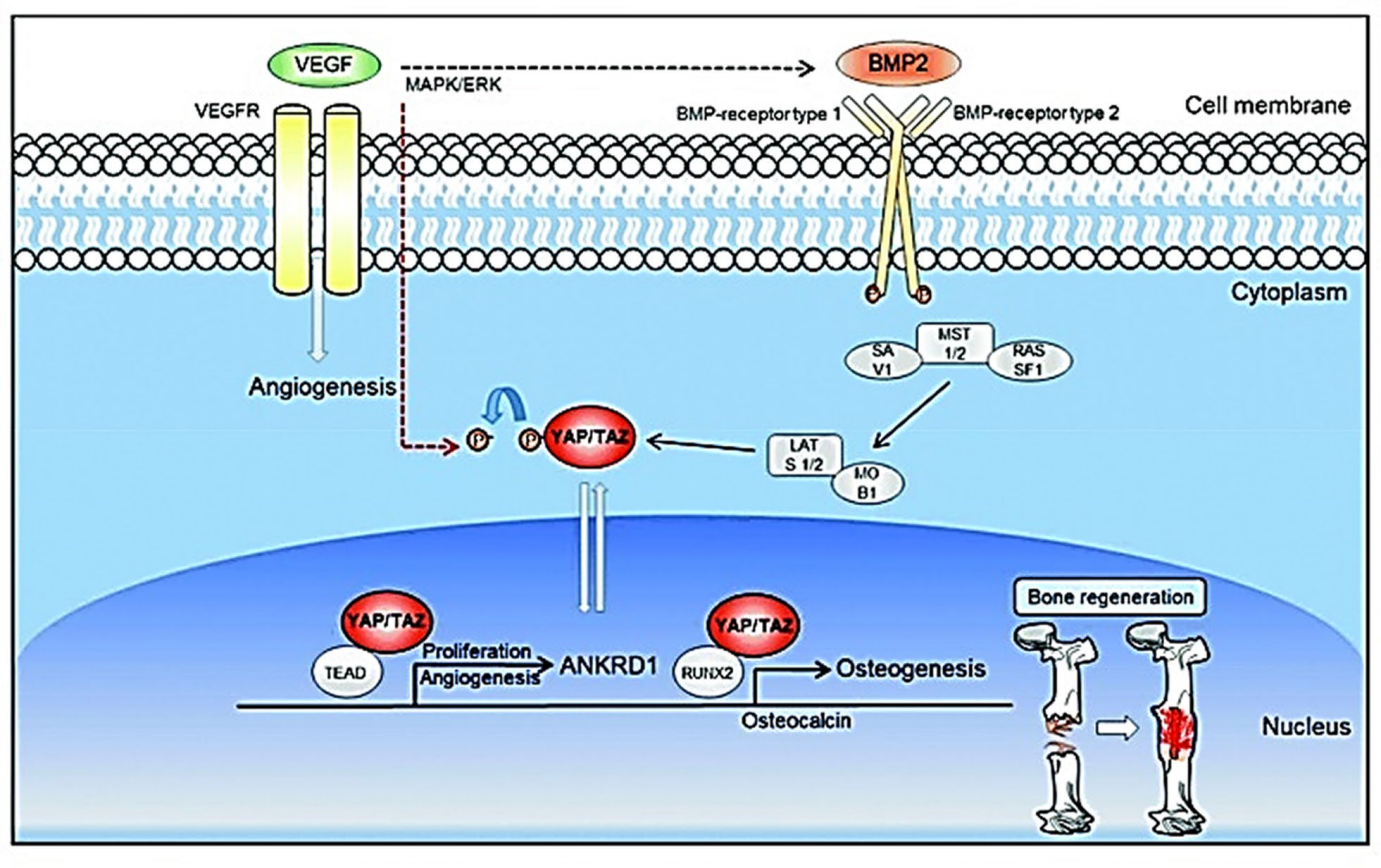
The synergistic relationship between genes that cause bone regeneration in adipose stem cells transfected with BMP2 and VEGF genes is depicted in this diagram. Reproduced from [127] with permission

pDNA is an extrachromosomal small circular DNA which is mostly found in bacterial cells and is constituted by an antibiotic resistant gene, a promoter, the gene of interest and the origin of replication (Fig. 5) [128]. In the context of BTE, pDNA has been used to genetically engineer MSCs which have emerged as one of the favorite cell types in BTE applications due to their immunomodulatory properties, the ability of homing to the injured tissue site and to secrete several trophic agents [128–131]. The potential of pDNA in bone regeneration has been shown through several studies. For example, Li et al*.* transfected AD-MSCs with pDNAs encoding for BMP-2 and VEGF which were subsequently administered to treat a critical size calvarial defect [132]. By conducting mechanistic studies, the authors were able to demonstrate that the resulting bone regeneration was activated through YAP/TAZ signaling pathway (Fig. 6) [132]. YAP and TAZ play key roles in the maintenance, self-renewal, lineage commitment and expansion of SCs. Also, they act as co-regulators of RUNX2 and the signal transducer and activator of transcription factor 3 which are important transcription factors in bone hemostasis.

**Fig. 7:**
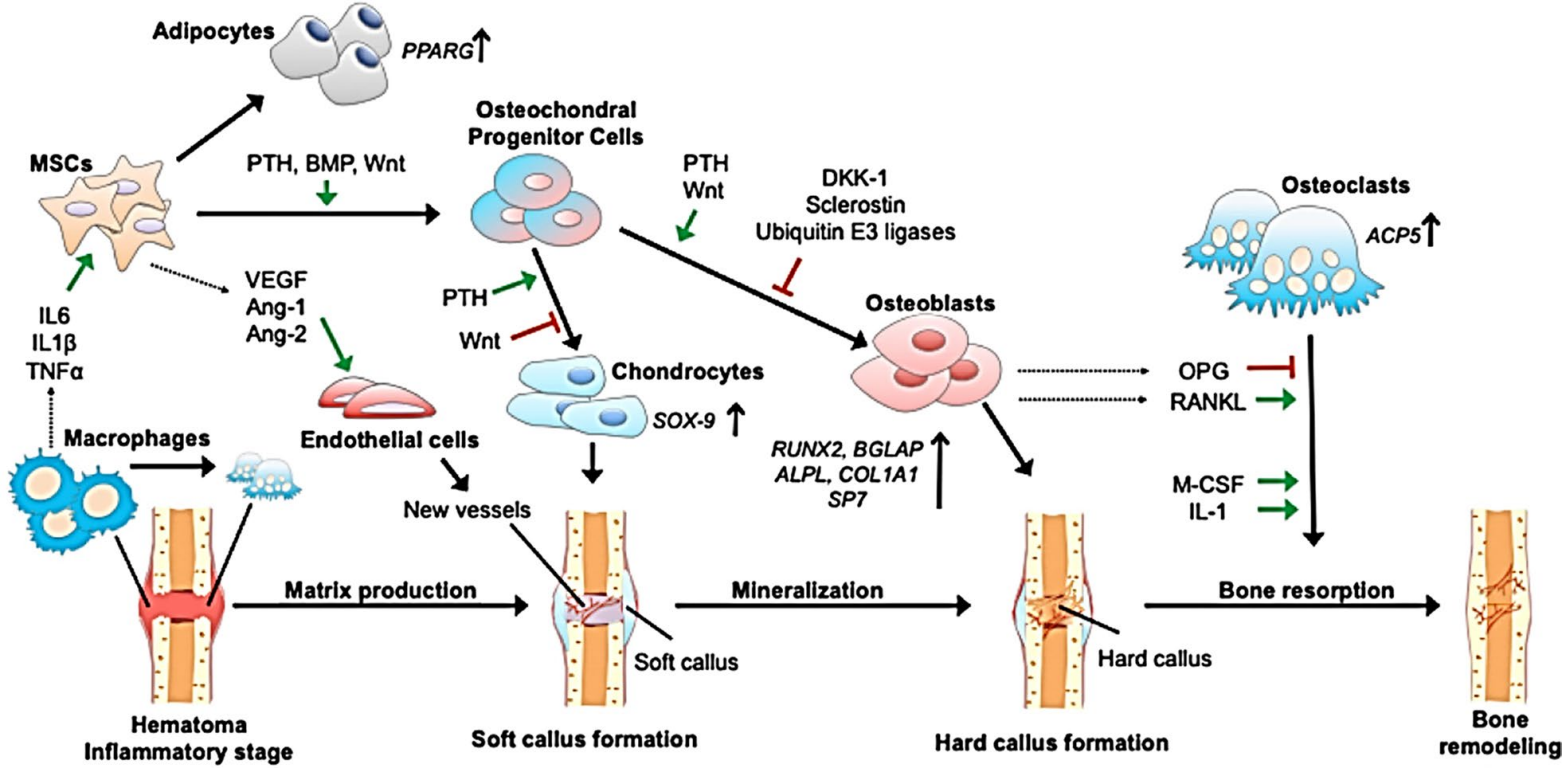
An illustration of fracture healing pointing out the different cells and factors that are either negative or positive regulators of regeneration, as well as potential targets for siRNA interventions. DKK1, dickkopf-related protein 1; OPG, osteoprotegerin; ALPL, alkaline phosphatase; PPARG, peroxisome proliferator-activated receptor gamma; Wnt, wingless-related integration site; M-CSF, macrophage colony-stimulating factor; ANG, angiopoietin; SOX-9, sex determining region Y-Box 9; ACP5, acid phosphatase 5; BGLAP, bone gamma-carboxyglutamate protein (Osteocalcin); MSC, mesenchymal stem cell; RANKL, receptor activator of nuclear factor kappa-B ligand; PTH, parathyroid hormone; RUNX2, runt-related transcription factor 2; TNFα, tumor necrosis factor alpha; VEGF, vascular endothelial growth factor. Reproduced from [135] with permission

Another approach to promote bone regeneration is to prevent the translation of a protein in the target cell which is done by the selective degradation of the target protein’s encoded mRNA [133]. For that, siRNA, which is a type of noncoding double stranded RNA which can act as a direct inhibitor of a specific protein, has been employed (Fig. 7). Examples include the work of Cui et al. which fabricated cationic sterosomes (which are a type of liposomes constituted by stearylamine and cholesterol) for the delivery of siRNA against noggin [134]. Being noggin a BMP antagonist, nogging knockdown by the siRNA entrapped within the sterosomes led to the osteogenic differentiation of AD-MSCs in vitro and bone regeneration in vivo.

miRNAs are also small noncoding RNA sequences which regulate gene expression through RNA interference [136]. However, in contrast to siRNAs, miRNAs possess several targets and do not specifically target one mRNA. miRNAs have been successfully employed to promote both angiogenesis and osteogenesis. For example, Zhang et al*.* transfected BM-SCs with miRNA 378 to promote enhanced ALP activity, mineralization and VEGF secretion [137]. Li et al. conducted another investigation where miRNA 26a was delivered to repair a CSBD mouse model. The results revealed that miRNA 26a was able to promote bone regeneration by concurrently enhancing vascularization and bone formation as shown by the up-regulation of four angiogenesis-osteogenesis-related genes (i.e., angiopoietin 1, VEGF, RUNX2 and OCN) [138]. Various research studies have consistently demonstrated that miRNA-7b, -9, -21, -26a, -27a, -210, -378, -195–497 cluster, -378, and -675 exert a favorable influence on both angiogenesis and osteogenesis [137, 139–142]. Conversely, miRNA-10a, -222, and -494 have been observed to impede these processes [136, 141, 143, 144]. These significant findings highlight the essential regulatory role played by these specific miRNAs in maintaining a delicate equilibrium between angiogenesis and osteogenesis.

Considering the potential of nucleic acid delivery to promote bone regeneration, research efforts have focused on developing the so-called gene-activated matrices as a strategy to circumvent the use of viral vectors. Successful gene delivery depends on developing efficient, nonimmunogenic delivery platforms which promote transfection efficiency and provide controlled delivery of the desired factors [26]. While viral vectors promote the highest transfection efficiency, their use is accompanied by serious concerns on safety issues such as insertional mutagenicity, small capacity for the transfer of the gene cargo, possible immunogenicity and difficulties in their production procedure and scale up [145–149]. In contrast, nonviral carriers have shown superior safety while being easier to scale up.

**Fig. 8:**
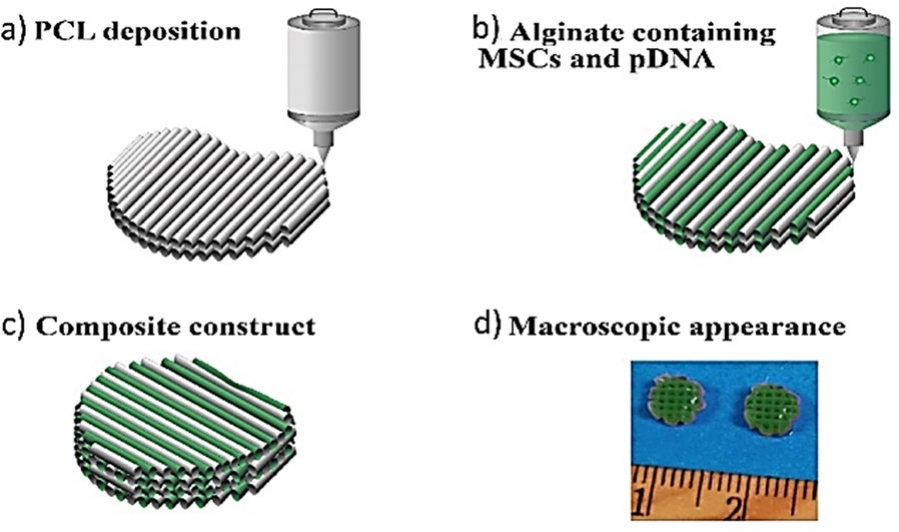
**a**–**c** bioprinting is shown in the following diagram, which shows gene activated bioink containing nHA-plasmid DNA complexes, alginate, and mesenchymal stem cells (MSCs) was loaded in the piston system for coprinting with poly caprolactone (PCL). Moreover, **d** the macroscopic appearance of the constructs is shown before implantation

**Fig. 9:**
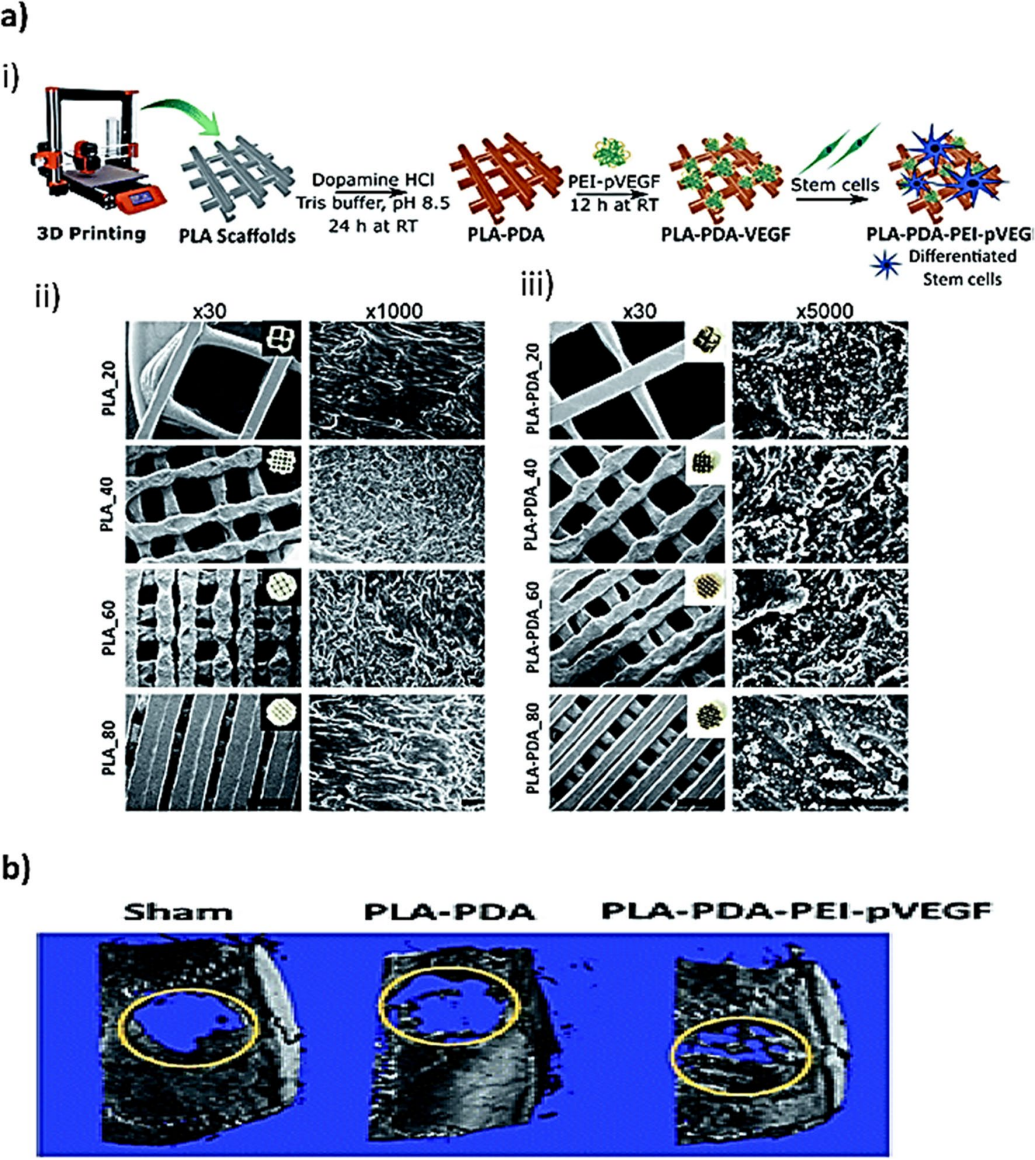
**a** This diagram illustrates a complete process of 3D printing functionalized PLA scaffolds coated with PEI-pVEGF. i) An overview of the process of generating scaffolds using 3D printing. ii to iii) SEM images of 3D printed PLA scaffolds with varying infill percentages. **b** An imaging microCT scan showing the regenerated bone in the yellow circle 30 days after implant scaffolds. The Royal Society of Chemistry has granted the permission to reproduce [153]

Thus, the use of gene-activated matrices which consist of scaffold platforms able to provide a suitable microenvironment for cellular attachment, proliferation and differentiation have emerged as a promising approach to concurrently facilitate the local transfection of the target cells, using non-viral vectors [26]. The first study on gene-activated matrices was reported as early as in 1996 by Fang et al*.* [150]. The authors used a collagen sponge to immobilize two pDNAs encoding the parathyroid hormone fragment and BMP-4, respectively, which worked synergistically in vitro and led to bone regeneration in vivo. A more recent study to produce efficient gene activated constructs, involves the work by Cunniffe et al*.* where pDNA encoding for BMP-2 and TGF-β was complexed to an RGD-linked alginate and nano-HA-based scaffold [151]. The resulting gene-activated bioink was co-printed together with a PCL microfilament together with MSCs (Fig. 8) [151]. The resulting gene-activated MSC-laden constructs were implanted subcutaneously in nude mice and the results revealed sustained protein expression for up to 14 days as well as the production of a vascularized and mineralized tissue following 12 weeks after implantation. On a different study, Gonzalez-Fernandez et al*.* incorporated a pDNA also encoding TGF-β3 and BMP-2 within nano-HA NPs which were subsequently embedded into an alginate scaffold. Their results demonstrated a sustained expression of the transgenes on MSCs cultured for 14 days. Interestingly, the as-prepared pDNA-containing scaffold was able to direct the fate of the MSCs towards either a chondrogenic or an osteogenic phenotype based on whether the BMP-2- and TGF-β3-encoding pDNAs were delivered separately or jointly [126]. Another study indicating the potential of such an approach was conducted by Raftery et al*.* [152]. The authors fabricated an HA and collagen scaffold platform incorporating CHI NPs carrying BMP-2 and VEGF-encoding pDNA genes. The resulting gene-activated scaffold was resorbable, biocompatible and also facilitated cellular adhesion, proliferation and differentiation. Also, sustained protein expression (i.e., BMP-2 and VEGF) was obtained for up to 28 days. Then, the scaffold was used to accelerate bone regeneration in a CSBD rat model. The results showed bone formation after only 28 days post implantation when the gene-activated matrix was used as compared to the 8 weeks needed when the free BMP-2 GF was administered [152]. On a follow up study, the same group evaluated the efficiency of a series of BMP-2-encoding pDNAs with different promoters to transfect MSCs. CHI NPs were also chosen as the carrier to encapsulate the pDNA and transfect the MSCs. The transfected MSCs were then embedded into a collagen-HA composite scaffold which was subsequently used in vivo in a CSBD model. The results confirmed that transfection of the BMP-2-encoding pDNA led to enhanced levels of BMP-2 expression, higher ALP activity and increased calcium deposition [15]. Chakka et al*.* fabricated a gene-activated scaffold platform made of poly(lactic acid) which was coated with polydopamine and surface decorated with polyethyleneimine (PEI) and VEGF-encoding pDNA (Fig. 9). Such a construct was evaluated in vivo in a rat calvarial critical bone defect model and the results showed improved vascularization which translated into significantly greater bone regeneration [153]. Another study using PEI involves the work by Khorsand et al. [154]. The authors fabricated nanoplexes of pDNA encoding for FGF and BMP-2 and PEI that were about 80–117 nm in size which were subsequently incorporated into a collagen scaffold. The results showed how the gene-activated scaffold could significantly increase bone regeneration in vivo using a diabetic rabbit model displaying diaphyseal long bone radial defects.

Taken together and as shown by these examples, the fabrication of gene activated scaffolds provides a platform for sustained gene expression and results in the localized production of the targeted proteins. This, in turn, enhances cellular adhesion, proliferation and differentiation and the resulting bone regeneration. However, despite the promise of such an approach, a main challenge that still remains to be addressed is to achieve good transfection efficiency of the non-viral vectors which display diminished immunogenicity and side effects as compared to the viral ones [155].

### Multifunctional scaffolds incorporating bioactive agents and cells

BTE makes it possible to create novel platforms that incorporate not only bioactive agents, but also different relevant cell lines needed for regenerating the lost bone tissue, rendering the so-called multifunctional scaffolds. Nevertheless, the development of bioactive porous scaffolds with multifunctional properties is still an important challenge. One approach to address this challenge is to combine the scaffold platform with the primary cell types found in natural bone. During bone regeneration, there are synergistic interactions between osteogenic and vasculogenic cells. These cell types play a crucial role in the bone regeneration process, making them essential for successful BTE. Consequently, we have introduced multifunctional platforms with the potential to significantly enhance the effectiveness of BTE strategies, ultimately leading to improved outcomes in the field of regenerative medicine.

#### Scaffolds incorporating cells

**Fig. 10:**
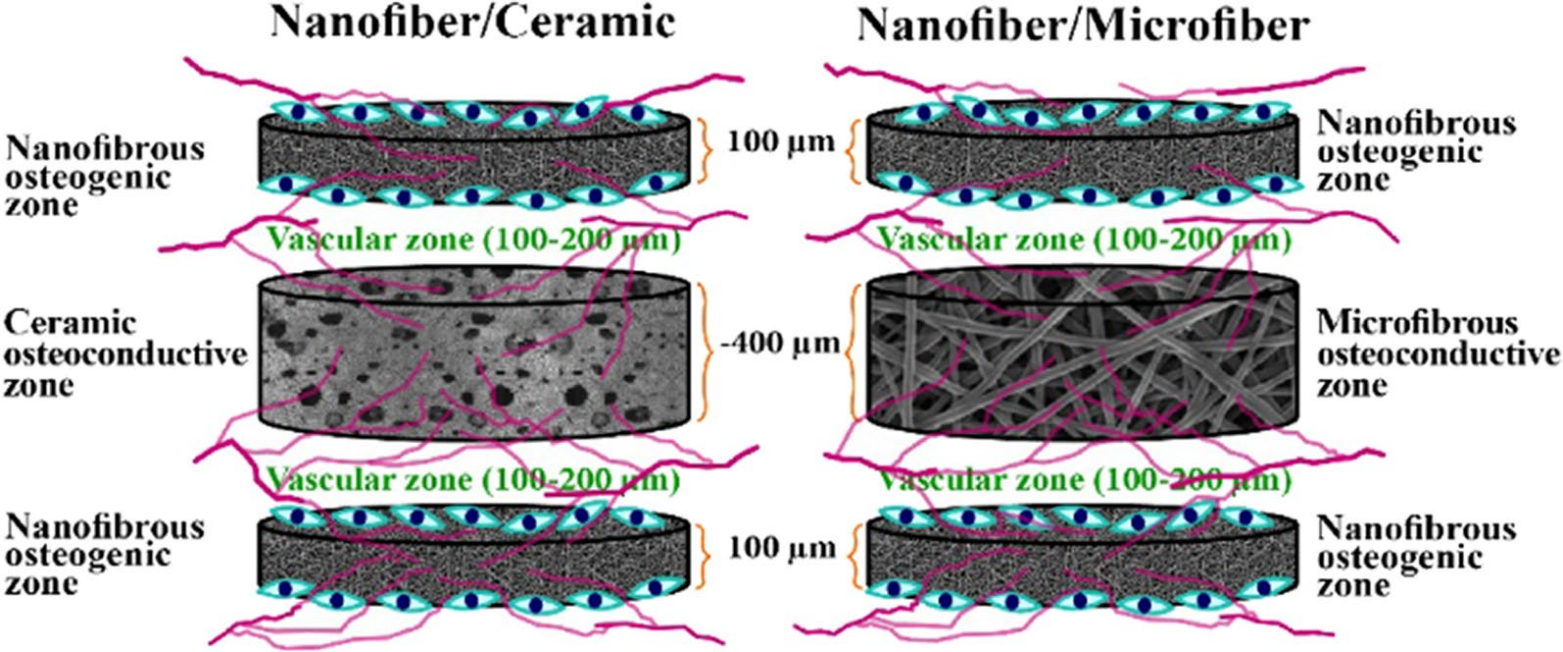
This schematic shows porous osteoconductive and nanofibrous osteogenic layers in microscale thickness, as well as suitable microenvironment for vascularization between the layers

One of the most relevant cell types used as a tool for bone regeneration are MSCs. MSCs can promote angiogenesis and bone regeneration by secreting biologically active molecules with low risk of immunogenicity. As such, it has been shown how, the coculture of MSCs and ECs on Ca-P scaffolds, led to the enhanced formation of capillary-like structures and promoted osteogenesis and mineralization in rats [156]. In another study, coculturing MSCs and EPCs synergistically induced angiogenesis and bone regeneration. This result was attributed to the ability of the cells to release soluble factors such as BMPs, TGF-β and VEGF [157]. Interestingly, the mechanical stimulation of MSCs can also induce both osteogenesis and angiogenesis. In this regard, Charoenpanich et al. showed that, by applying a uniaxial cyclic tensile strain to a scaffold-containing MSCs, it was possible to induce osteogenesis and angiogenesis as shown by microarray analysis [158]. This is due to the ability of the mechanical stimulus to modulate five relevant genes in bone remodeling, namely, BMP-1 (from BMP signaling), PIK3CD (from PIKs signaling), FZD8 (Frizzled), WNT5B and the transcription factor TCF4 (T-cell factor). This, in turn, translates in the regulation of the Wnt/BMP/PIK signaling pathway which promotes osteogenesis by increasing the expression and activation of RUNX2, Dlx5 and Osterix. On a very advanced design, Sathy et al*.* combined MSCs together with HUVECs and pericytes in a single platform [159]. For that, the authors designed a multilayered scaffold by alternating microscale-thick layers of MSCs-containing nanofibers (as the osteogenic layer) with microfibers or porous ceramics (as the osteoconductive layer). At the interfaces, a HUVEC- and pericytes-containing gel composed by collagen and fibronectin as the angiogenic zone was also incorporated (see Fig. 10 for details). The best construct was the one in which the angiogenesis zone was designed at the interface of the osteoconductive and the osteoinductive layers. The layered constructs were inserted in mice and the regeneration of bone tissue and vascularization were followed by histological analysis. Their results indicated that vascularization took place through the angiogenic zone which also affected the mineralization of the constructs.

#### Scaffolds incorporating multiple bioactive agents

**Fig. 11:**
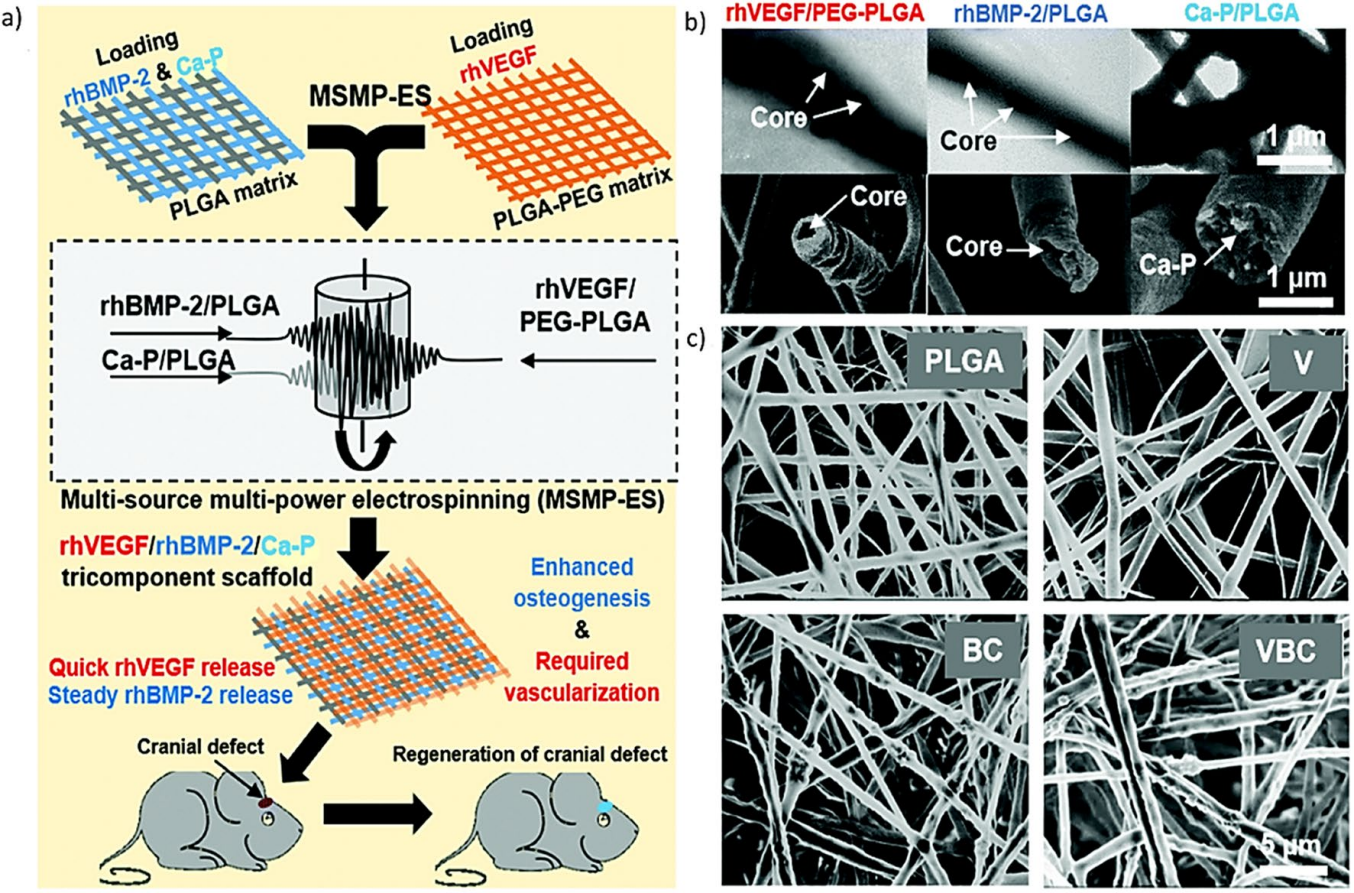
Schematic of tricomponent scaffold: **a** fabrication, sequential release of GFs and implantation into the mice with cranial defects. **b** Tricomponent scaffolds with fibrous components are shown in SEM and TEM micrographs. **c** SEM micrographs of tricomponent scaffolds and control groups (PLGA, BC, VBC and V scaffolds). Reproduced from [80] with permission

Another approach to create multifunctional scaffolds is by incorporating multiple active agents [160, 161]. As an example, a three-component scaffold was reported Wang et al. who fabricated electrospun fibers incorporating rhBMP-2, rhVEGF and Ca-P NPs. The fragile functional components were first mixed with PLGA-PEG (for rhVEGF) and PLGA (for rhBMP-2 and Ca-P) and the fibers were created via emulsion electrospinning (Fig. 11) [80]. Next, two different cell lines (i.e., HUVECs and hBM-MSCs) were pre-seeded onto the scaffolds and the results indicated increased proliferation of HUVEC, tube formation and CD31 expression combined with better osteogenic differentiation [80].

**Fig. 12:**
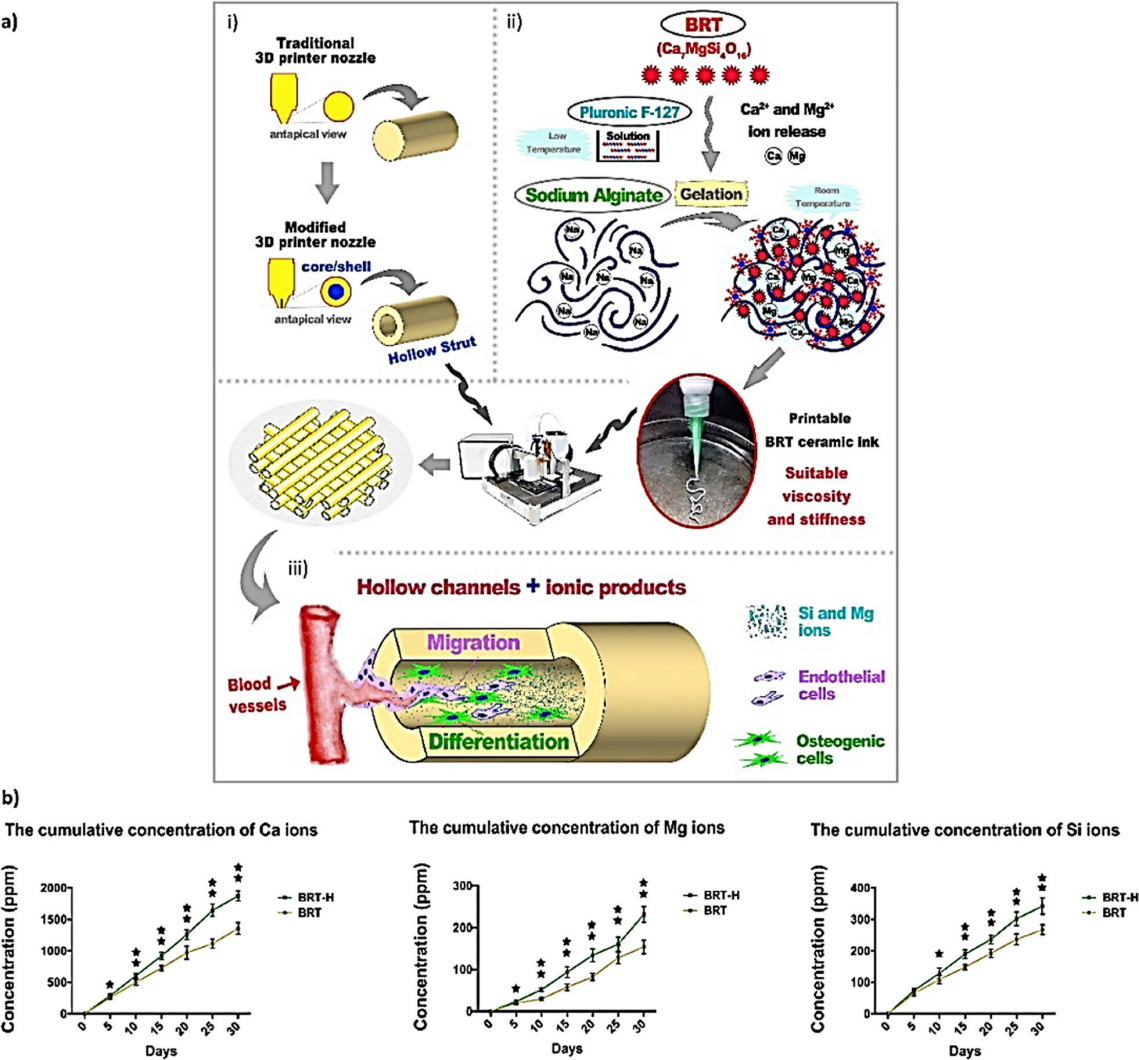
**a** Utilizing the synergistic effect of bioactive ions and pipeline structure for vascularized bone regeneration in 3D printed bioceramics scaffolds (i to ii) The modification of the printer nozzle with a core shell structure and a bioceramic paste with viscoelasticity. iii) An illustration of the printing process for hollow BRT (BRT-H) pipes through the synergistic effect of bioactive ions and pipeline structures. **b** Ionic release from 3D printed bioceramics scaffold (BRT) and BRT-H for 30 days. Reproduced from [162] with permission

In the context of scaffolds entrapping multiple active compounds, it is of special relevance to achieve a spatiotemporal control over their release. By doing so, the local regenerative process of the bone can be accelerated and undesired systemic effects mitigated. Controlled release of osteogenic and angiogenic factors has been achieved by several groups. For example, Wang et al. fabricated a 3D printed scaffold composed of an osteogenic peptide, β-TCP and PLGA which was subsequently coated by collagen I and an angiogenic peptide [81]. This dual peptide structure led to a fast release of the angiogenic peptide while the osteogenic one was released in a sustained and slow manner. The in vivo results revealed that the sequence release patterns improved bone formation through induction of both angiogenic and osteogenic properties. Another example where controlled release was achieved using a multifunctional scaffold was reported by Zhang et al. [162]. The authors fabricated a hollow-pipe-packed bioceramic scaffold platform using coaxial 3D printing. The as-prepared scaffold was constituted by sodium alginate and Pluronic F127 as the organic components and silicate dopped by Mg^2+^ and calcium ions (Ca^2+^) (i.e., Ca_7_MgSi_4_O_16_) as the inorganic one (Fig. 12). Their results showed enhanced blood vessel formation within the inner part of the hollow pipes which was attributed to a fast Ca^2+^, Mg^2+^ and SiO_4_^2−^ release while the silicate coating could effectively enhance bone tissue formation [163]. This is not surprising since previous studies have shown the ability of both Mg^2+^ and SiO_4_^2−^ to enhance osteogenesis and also angiogenesis by promoting the crosstalk between the ECs and the bone marrow stromal cells [163, 164]. On a different study, the release of the bioactive compounds was controlled by making use of nanocarriers. In this regard, Alarcin et al*.* developed a shear thinning hydrogel constituted by gelatin and silicate nanoplatelets also incorporating rhVEGF-loaded PCL/poly(vinyl alcohol) (PVA) NPs [165]. The results showed that the PCL/PVA NPs enhanced the entrapment efficiency of the GF and led to a sustained release pattern over a period of 7 days. Dyondi et al. used CHI NPs for the incorporation of BMP-7 and bFGF into gellan xanthan gels [166]. The release pattern study demonstrated that the GFs were released in a sustained manner when incorporated into the NPs. By doing so, the burst release was significantly diminished and, probably, by this strategy, the cost of the treatment will also be reduced since smaller amounts of GFs are needed. What is more, the possible adverse effects associated with using large amounts of GFs could also be diminished [166].

Table 4 provides a comprehensive summary of bioactive compounds that have been utilized either through carrier systems or by direct incorporation into scaffolds, as discussed within the article. These compounds demonstrate the potential to effectively promote both angiogenesis and osteogenesis simultaneously.

**Table 4:** A summary of the utilization of bioactive compounds incorporated into functional scaffolds to promote both osteogenesis and angiogenesis

Bioactive compound	Scaffold material	Characteristic	References
VEGF and BMP-2	Mesoporous BGs, sulfated CHI GelMA hydrogel	Faster release of VEGF and sustained and slower release of rhBMP-2 promoted capillary tube formation and osteogenic differentiation	[14]
TUDCA and BMP 2	Nanofibrous PLA	Burst release of TUDCA and sustained release for BMP-2 promoted enhanced osteogenesis and angiogenesis	[74]
Salvianolic acid B	PLGA and β-TCP	Controlled release of salvianolic acid B promoted bone fusion through angiogenesis and osteogenesis in a rat spinal fusion model	[167]
DEX loaded Ca-P -based NPs	Collagen	DEX promoted osteogenesis and the scaffold’s microgroove network promoted the alignment of HUVECs into tubular structures and resulted in rapid angiogenesis	[103]
rhBMP-2 and rhVEGF	PLGA and EG	Rapid release of rhVEGF and controlled release of rhBMP-2 promoted osteogenic differentiation and the formation of tubes	[80]
VEGF	PLGA and fibrin	Activation of BM stromal cells leading to the generation of new vessels. Enhanced the release of osteogenic factors, enhancing fracture healing	[32]
FGF-1	Fibrin and HA	Enhanced angiogenesis and stimulating the infiltration of cells expressing osteogenic markers	[40]
Nanosilicate and DMOG	PLGA nanofibers	Enhancement and orchestration of the osteogenesis-angiogenesis processes	[105]
BMP-2 and VEGF	PCL and HA	Rapid VEGF release and gradual sustained release of BMP-2 promoted both angiogenesis and osteogenesis	[54]
Simvastatin and DEX	PCL and collagen	Enhanced osteogenic differentiation and tube formation over a period of 21 days	[70]
FGF-2, BMP-2 and VEGF	Silica coated nano HA-gelatin reinforced with electrospun PLA yarns	Enhanced effect on angiogenesis and bone formation was noticed	[71]
DFO	PCL and carboxymethyl CHI	Enhanced vascularity regeneration leading to more bone formation and osseointegration	[93]
Exosomes derived from hAD-SCs	PLGA-Mg^2+^-GA	The sustained release of Mg2+, GA, and exosomes from the scaffolds for up to 10 days was capable of inducing osteogenesis and angiogenesis	[122]
BBG	PCL	Release of Ca^2+^, Na^+^, and BO_43−_, thereby promoting cellular proliferation and enhancing osteoblastogenesis and angiogenesis	[168]
pDNA encoding for FGF and BMP-2	Collagen and PEI	Augmented the angiogenesis and osteogenesis	[154]
pDNA encoding for BMP-2 and TGF-β	Nano-HA	vascularization and mineralization in a subcutaneous environment	[151]
VEGF-encoding pDNA	PLA and PDA	Augmented VEGF and BMP-2, promoting angiogenesis and enhancing osteogenic differentiation	[153]
CHI NPs, BMP-2 and VEGF-encoding pDNA	HA and collagen	Synergistic effect, inducing both osteogenesis and angiogenesis	[152]

CHI, chitosan; VEGF, vascular endothelial growth factor; DEX, dexamethasone; BMP, bone morphogenetic proteins; NPs, nanoparticles; pDNA, plasmid DNA; PLA, poly lactic acid; PDA, polydopamine; PEI, polyethyleneimin; TGF-β, transforming growth factor; HA, hydroxyapatite; β-TCP, β-Tricalcium phosphate; PCL, polycaprolactone; GA, gallic acid; DFO, deferoxamine; DMOG, dimethyloxalylglycine; PLGA, poly lactic-*co*-glycolic acid; FGF, fibroblast growth factors; GelMA, gelatin-methacryloyl; BM, bone marrow; TUDCA, tauroursodeoxycholic acid; Ca-P, calcium phosphate; hAD-SCs, human adipose-derived stem cells; BBG, borate bioactive glasses; HUVECs, human umbilical vein endothelial cells

## Exploring biocompatibility and the degradation fate of scaffolds

In recent years, efforts have been made to enhance the characteristics of bone biomimetic scaffolds for clinical trials. When evaluating these scaffolds in BTE, two important factors should be considered: biocompatibility and degradation profile. Biocompatibility determines how well a material can function in a biological environment without causing negative reactions, while the degradation profile of a scaffold should ideally allow for synchronized tissue regeneration and non-toxic metabolization or excretion of degradation products [169].

Currently, a variety of scaffolds, including natural and synthetic polymers, BG-based scaffolds, and bioceramics have shown promise in promoting BTE. The biocompatibility and biodegradability of natural polymers like collagen, CHI, hyaluronic acid, gelatin and alginate have made them pivotal in tissue regeneration research. However, the rate of degradation for these natural polymers depends on the enzyme activity found in different patients [170]. Collagen, for instance, has been identified in at least 28 different types [171]. Furthermore, the deacetylation degree of CHI affects its biodegradability, with CHI scaffolds above 71.7% deacetylation degrading slowly and below 71.7% degrading rapidly [172]. Similarly, the concentration of alginate influences the degradation of alginate derivative gels, with lower concentrations degrading at a much faster rate compared to those containing a higher concentration [173]. It should be noted that, while natural polymers derived from human or animal sources, may yield positive results in in vitro compatibility tests, they can also trigger adverse immune reactions or disease transmission. Collagen-based scaffolds, for example, possess desirable qualities but can elicit different immune responses due to factors such as allergies, foreign body reactions, and species differences between donor and recipient [174].

The commonly used synthetic polymers in BTE include PCL, PLGA, PVA, PGA, PLA, and PEG. PLA, PGA, and PLGA have been approved by the FDA for clinical use. Synthetic polymer breakdown products usually have mild acidity, and excessive acidity can hinder cell growth on bone biomaterials and potentially cause inflammation [175]. Clinical studies show that unspecific inflammation resulting from PLA and PGA can occur at a rate of up to 8% [176]. PCL has a semi-crystalline structure and hydrophobic properties, leading to a slow degradation process in the body that can last for multiple years, making it suitable for scaffold applications [175]. On the other hand, PVA has beneficial hydrophilic properties, excellent chemical stability and can degrade in the body without causing harm to human health [175].

There has been significant advancement in the development of bioceramics, with BG being extensively utilized for bone regeneration purposes. BG, a subgroup of bioceramics, possesses remarkable regenerative properties. These glasses consist of SiO_2_, calcium oxide (CaO), and sodium oxide (Na_2_O), gradually degrading over time and releasing beneficial ions for bone nutrition and formation. The biocompatibility and cytotoxicity of BG, especially those with silica, depend on administration method and dosage [175, 177]. TCP, biphasic Ca-P, and HA are widely used bioceramics in biomimetic scaffolds due to their favorable biocompatibility, biodegradability, osteoconductivity, and osteoinduction. These bioceramics actively participate in human metabolism, creating an alkaline environment that enhances cell activity and accelerates bone repair [175]. HA has high crystallinity and stability, while TCP has a faster degradation rate, being 10–20 times faster than HA [178].

However, further investigation is imperative to fully comprehend the influential factors impacting the immune response. Furthermore, the utilization of biocompatible tracing methods and substances is pivotal to effectively monitor the degradation process of scaffolds.

## Clinical studies

In 2006, a first clinical trial was conducted (NCT00310440) where 319 patients were recruited to assess the effectiveness and safety of administering a bone putty containing P-15 (which is a collagen-derived peptide that promotes stem cell differentiation and adhesion) into a local autologous bone defect [179]. In 2016, the FDA issued the first premarket approval for the I-FACTOR™ product. Such a product is provided to the clinician in the form of a prefilled syringe with the graft material [179]. Notably, a phase III clinical investigation is currently ongoing to assess the efficacy of the gene activated matrix known as Nukleostim in the maxillofacial region (NCT02293031). Nukleostim is composed of a collagen-HA composite scaffold and DNA plasmids containing VEGF-A165. As compared to placebo treatment, Neovasculgen® (composed of a plasmid DNA encoding VEGF 165) doubled pain-free walking distance in patients with critical limb ischemia [26, 180, 181].

Over the past decade, many preclinical but also clinical studies have demonstrated the value of employing MSCs in conjunction with various types of scaffolds for bone tissue regeneration. In this context, the majority of clinical trials combined MSCs with HA, TCP, Ca-P and demineralized bone tissue and collagen (as shown in Table 5). Even though in vitro experiments have shown that cell-based constructs could effectively be used in bone regeneration, the results of very few clinical trials on cell-based bone constructs have been published to date. Unfortunately, investigators do not always have easy access to clinical trial data, and most of these outcomes are not published in peer-reviewed journals.

**Table 5:** List of clinical studies using MSCs combined with biomaterials for bone tissue regeneration listed in clinicaltraisl.gov

Bone defect	Cell types	Sponsor	NCT number	Phase	Patient	Scaffold
Maxillary cyst	Mesenchymal stem cell	Red de terapia celular	NCT01389661	2	11	BioMax (a serum cross-linked scaffold)
Periodontal intrabony defects	Amnion membrane	Krishnadevaraya College of Dental Science	NCT02635529	4	10	Deproteinized bovine bone mineral and collagen membrane
Periodontal disease	Dental pulp stem cell	University of Turine	NCT03386877	Not applicable	29	Collagen scaffold
Cleft lip and palate	Dental pulp MSCs	Hospital Sirio-libanse	NCT03766217	3	6	HA and collagen composite scaffold
Osteochondritis	MSCs	Istituto Ortopedico Rizzoli	NCT02005861	Not applicable	140	Collagen scaffold
Cleft lip and palate	MSCs	Cairo University	NCT03563495	Not applicable	10	Collagen scaffold
Osteonecrosis jaw	MSCs	Red de terapia celular	NCT02566681	1	10	TCP matrix and demineralized bone matrix

MSCs, mesenchymal stem cells

## Conclusion and future prospect

The growing elderly population all over the world and the limitations associated with current bone graft substitutes have made researchers focus on improving BTE methods. Despite the progress in developing bone graft substitutes, fabrication of constructs with clinical relevance has become a significant issue and the complex design of the bone has resulted in limited efficiency for the current available methods.

The design and fabrication of functional bone graft substitutes requires a wide knowledge of the ECM of the natural bone, the precise mechanism associated with the healing procedure of the bone tissue and the recent developments on new technologies. To this end, a detailed and organized approach towards designing a multifunctional scaffold made of appropriate biomaterials, specific structures and various cell lines regulating the sequential release of GFs and other bioactive agents seems to be essential.

One of the most significant difficulties in the development of multifunctional scaffolds is the vascularization of the neo-tissue which is critical in nourishing the central parts of the engineered tissue. There are several approaches to meet the aforementioned challenge. The direct incorporation of GF-loaded NPs into the scaffold, the use of gene activated matrices or the incorporation of combinatory vasculogenic/osteogenic cells into the scaffold platforms are some of the solutions discussed in this review. Moreover, the design and fabrication of scaffold platforms with adequate mechanical stability, porosity, suitable surface topography will take place only by making use of recent pioneering fabrication technologies. Besides, such technologies also make it possible to tailor the scaffold properties according to the patient’s essentials through computer-aided manufacturing and computer-aided design. In conclusion, we believe that the designing and fabrication of biomimetic scaffold platforms is an interdisciplinary issue which needs close collaboration of clinicians, engineers and biomaterial scientists to reach the final goal.

## Data Availability

Not applicable.

## Abbreviations

AD-MSCs: Adipose derived mesenchymal stem cells
ALP: Alkaline phosphatase
AM: Additive manufacturing
BG: Bioactive glass
BM: Bone marrow
BMPs: Bone morphogenetic proteins
BTE: Bone tissue engineering
Ca2+: Calcium ions
CAD: Computer-aided design
Ca-P: Calcium phosphate
CHI: Chitosan
CMC: Carboxymethyl chitosan
CSBD: Critical sized bone defects
DMOG: Dimethyloxaloylglycine
ECs: Endothelial cells
ECM: Extracellular matrix
EPCs: Endothelial progenitor cells
FDA: Food and Drug Administration
FDM: Fused deposition modeling
FGF: Fibroblast growth factors
GelMA: Gelatin-methacryloyl
GF: Growth factor
HA: Hydroxy apatite
hAD-SCs: Human adipose-derived stem cells
hBM-MSCs: Human bone marrow-derived mesenchymal stem cells
HIF-1: Hypoxia-inducible factor 1
hiPSCs: Human-induced pluripotent stem cell
HUVEC: Human umbilical vein endothelial cells
HIMC: Hierarchical intrafibrillarly mineralized collagen
IGF: Insulin-like growth factors
IL: Interleukin
LPN: Laponite
miRNA: Micro RNA
MSCs: Mesenchymal stem cells
MSMP-ES: Multi-source multi-power electrospinning
MSN: Mesoporous silica nanoparticles
NF-κB: Nuclear factor-kappaB
NPs: Nanoparticles
OBs: Osteoblasts
OCN: Osteocalcin
OP: Osteogenic peptide
OPN: Osteopontin
PCL: Poly caprolactone
PDA: Polydopamine
PDGF: Platelet-derived growth factors
pDNA: Plasmid DNA
PEG: Poly(ethylene glycol)
PEI: Polyethyleneimine
PEKK: Polyetherketoneketone
(PI3K/AKT: Phosphatidylinositol 3 kinase (PI3K)/protein kinase B (AKT)
PLLA: Poly(l-lactic acid)
PLA: Poly(lactic acid)
PLGA: Poly lactic-*co*-glycolic acid
PVA: Poly(vinyl alcohol)
PHBV: Poly(hydroxybutyrate-*co*-hydroxy valerate)
rh: Recombinant human
RUNX2: Runt-related transcription factor 2
SA: Stearylamine
SCs: Stem cells
siRNA: Small interfering RNA
Si^4+^: Silicon ions
SiO_2_: Silica
Sr^2+^: Strontium ions
SLA: Stereolithography
SLS: Selective laser sintering
STAT3: Signal transducer and activator of transcription factor 3
TCP: Tricalcium phosphate
TIPS: Thermal-induced phase separation
TGF-β: Transforming growth factor β
3D: Three-dimensional
TNF-α: Tumor necrosis factor-α
TUDCA: Tauroursodeoxycholic acid
VEGF: Vascular endothelial growth factors
